# Adaption of neurosurgical resection patterns for pediatric low‐grade glioma spanning two decades—Report from the German LGG‐studies 1996–2018

**DOI:** 10.1002/cam4.7417

**Published:** 2024-06-24

**Authors:** Tibor Kelety, Ulrich‐Wilhelm Thomale, Daniela Kandels, Martin U. Schuhmann, Ahmed El Damaty, Jürgen Krauss, Michael C. Frühwald, Pablo Hernáiz Driever, Olaf Witt, Brigitte Bison, Monika Warmuth‐Metz, Torsten Pietsch, René Schmidt, Astrid K. Gnekow

**Affiliations:** ^1^ Pediatrics and Adolescent Medicine, Swabian Children's Cancer Center University Hospital Augsburg Augsburg Germany; ^2^ Pediatric Neurosurgery, Campus Virchow Klinikum, Charité – Universitaetsmedizin Berlin Berlin Germany; ^3^ Division of Pediatric Neurosurgery, Department of Neurosurgery University Hospital of Tuebingen, Eberhard Karls University Tuebingen Germany; ^4^ Division of Pediatric Neurosurgery, Department of Neurosurgery Heidelberg University Heidelberg Germany; ^5^ Department of Pediatric Neurosurgery University Children's Hospital, University of Würzburg Würzburg Germany; ^6^ Department of Pediatric Oncology and Hematology Charité – Universitaetsmedizin Berlin, corporate member of Freie Universität Berlin and Humboldt Universität zu Berlin Berlin Germany; ^7^ Hopp Children's Cancer Center Heidelberg (KiTZ), Clinical Cooperation Unit Pediatric Oncology, German Cancer Research Center (DKFZ) Heidelberg University Hospital, National Center for Tumor Diseases (NCT) Heidelberg Germany; ^8^ Diagnostic and Interventional Neuroradiology, Faculty of Medicine University of Augsburg Augsburg Germany; ^9^ Institute of Diagnostic and Therapeutic Neuroradiology, University Hospital Würzburg Würzburg Germany; ^10^ Department of Neuropathology and DGNN Brain Tumor Reference Center University of Bonn Medical Center Bonn Germany; ^11^ Institute of Biostatistics and Clinical Research, University of Muenster Muenster Germany

**Keywords:** child, extent of resection, low‐grade glioma, neurosurgery, treatment algorithm

## Abstract

**Introduction:**

Neurosurgery is considered the mainstay of treatment for pediatric low‐grade glioma (LGG); the extent of resection determines subsequent stratification in current treatment protocols. Yet, surgical radicality must be balanced against the risks of complications that may affect long‐term quality of life. We investigated whether this consideration impacted surgical resection patterns over time for patients of the German LGG studies.

**Patients and Methods:**

Four thousand two hundred and seventy pediatric patients from three successive LGG studies (median age at diagnosis 7.6 years, neurofibromatosis (NF1) 14.7%) were grouped into 5 consecutive time intervals (TI1‐5) for date of diagnosis and analyzed for timing and extent of first surgery with respect to tumor site, histology, NF1‐status, sex, and age.

**Results:**

The fraction of radiological LGG diagnoses increased over time (TI1 12.6%; TI5 21.7%), while the extent of the first neurosurgical intervention (3440/4270) showed a reduced fraction of complete/subtotal and an increase of partial resections from TI1 to TI5. Binary logistic regression analysis for the first intervention within the first year following diagnosis confirmed the temporal trends (*p* < 0.001) and the link with tumor site for each extent of resection (*p* < 0.001). Higher age is related to more complete resections in the cerebellum and cerebral hemispheres.

**Conclusions:**

The declining extent of surgical resections over time was unrelated to patient characteristics. It paralleled the evolution of comprehensive treatment algorithms; thus, it may reflect alignment of surgical practice to recommendations in respect to age, tumor site, and NF1‐status integrated as such into current treatment guidelines. Further investigations are needed to understand how planning, performance, or tumor characteristics impact achieving surgical goals.

## Graphical abstract

Analyzing the pattern of the first neurosurgical intervention for 4270 German patients with pediatric LGG revealed significant changes over 22 years of recruitment in three consecutive studies: an increasing fraction of radiological LGG diagnoses, a decline in complete/subtotal resections at all tumor sites, an increase of partial resections, and a constant fraction of biopsies. With consistent epidemiologic data, these changes may be attributed to the growing acceptance of comprehensive treatment algorithms respecting age, tumor‐site, and NF1 status.

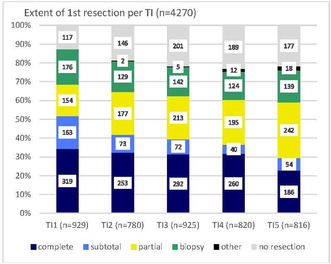

## 1 INTRODUCTION

Low‐grade glioma (LGG) account for almost half of all brain tumors in children and adolescents.^1^ They have long been described as slow‐growing tumors with heterogeneous biologic behavior but excellent long‐term survival. In recent years, molecular genetic diagnostics mapped distinctive mutations to various histologic subgroups,^2^ though most current treatment recommendations still comprise all pediatric LGG (pLGG) and few protocols stratify for molecular signatures.^3^

Neurosurgery is considered the mainstay of treatment for pLGG, with a role for diagnosis, reduction of mass effect, and tumor removal.^4^ Although complete resection emerged as favorable prognostic factor,^5^, ^6^, ^7^, ^8^ the risks of extended surgical resection for associated neurologic damage were acknowledged and caused debate for the choice of the optimal resection strategy at least for specific tumor sites like the supratentorial midline (SML).^9^, ^10^ Though key drivers for tumor regrowth or relapse and patterns of behavior of the various tumor subtypes are still not fully understood, size and site of tumor remnants play a significant role^5^, ^11^ giving neurosurgeons the challenge to minimize residuals. Over time, comprehensive treatment algorithms defined indications and timing for non‐surgical treatment for pLGG of all central nervous system (CNS) sites and histologies.^6^, ^12^, ^13^, ^14^, ^15^, ^16^ Still, the extent of surgical resection represented the pivotal point for subsequent treatment stratification. Following international consensus,^17^ tumor resection was even discouraged for intrinsic growth patterns and eloquent sites stressing that surgical radicality needed to be balanced against the risks of complications in order to preserve long‐term quality of life.

Successive multicenter studies for pediatric low‐grade glioma in Germany had been accompanied by a doubling of annual recruitment over two decades.^1^ A concurrent phenomenon was a relative decrease of complete/subtotal tumor removal at first surgery over the successive study periods. At first glance, it seemed counterintuitive that the rate of patients with complete primary resection decreased within 22 years, while modern neurosurgical management was increasingly supported by technical advances.^18^, ^19^, ^20^

Therefore, we analyzed surgical resection patterns in further detail for all patients registered in the HIT‐LGG 1996 study,^7^ the SIOP‐LGG 2004 study,^8^ and the LGG‐registry.^1^ We related the extent of primary resection over time to anatomical location, age at initial surgery, sex, NF1‐status, and histological diagnosis. We asked if reductions of the initial degree of resection correlated to changes of such epidemiologic characteristics within the study cohorts or other factors and affected radiological outcome.

## 2 PATIENTS AND METHODS

### 2.1 Patients

We included all study patients with pediatric LGG from the population‐based German cooperative multicenter studies HIT‐LGG 1996, SIOP‐LGG 2004 (ClinicalTrials.gov NCT00276640, EudraCT number 2005‐005377‐29), and LGG‐registry. Inclusion criteria comprised LGG of all CNS localizations in patients aged <18 years (<16 years prior amendment of April 27, 2007), histologic diagnosis of LGG according to the respective World Health Organization (WHO) classification, and no prior treatment. Central review for neuropathology and neuroradiology was recommended. Follow‐up included information up to August 4, 2020.

The registration period was segmented into five time intervals (TI) with comparable patient numbers for analysis of the extent of initial surgical resection over time. Time interval 1 (TI1) corresponded to the HIT‐LGG 1996 study period (01.10.1996 to 31.03.2004), intervals 2 and 3 (TI2, TI3) matched the first and second half of the SIOP‐LGG 2004 study period (01.04.2004 to 31.03.2012), and intervals 4 and 5 (TI4, TI5) the first and second half of the LGG registry period (01.04.2012 to 31.12.2018), respectively. Patients were allocated to these intervals according to the date of their tumor diagnosis, even if their study assignment differed.

### 2.2 Treatment guidelines

The basic treatment algorithm was identical throughout the entire study period and recommended for all pLGG patients. Following complete resection or incomplete removal without clinically symptomatic or progressive tumor remnants, patients were to be observed. Non‐surgical treatment was indicated for patients with residual tumor and neurological deterioration or continuous tumor progression in whom (re‐)resection was deemed unfeasible. Treatment of recurrence or progression was not standardized but included all modalities upon discussion in the local and/or national reference multidisciplinary tumor boards.

### 2.3 Neurosurgical treatment

All primary tumor‐related interventions were included in the analysis, also comprising those beyond adolescence. Despite protocol guidance, neurosurgical interventions cannot be standardized. Thus, decision‐making with respect to the extent of resection and timing may have varied during the study period and among participating centers.

The HIT‐LGG 1996 protocol recommended “maximal tumor resection or mass reduction” and avoiding risks for post‐surgical neurological impairments. Open or stereotactic biopsy should be performed in non‐resectable tumors to prove histology of LGG. During the SIOP‐LGG 2004 study and LGG registry, “best safe surgical excision” was recommended at diagnosis or relapse, but long‐term neurological/ophthalmological risks were emphasized to a greater extent, and disease stability following incompletely resected tumors was accepted as surgical goal.

The extent of the initial tumor‐related neurosurgical intervention was rated by the neurosurgeon during and following the intervention and by radiological findings based upon early postoperative scanning within 48–72 h, though categories were primarily defined radiologically as biopsy, partial, subtotal, or complete resection. Extent of resection was complete if there was no evidence of residual tumor, and as subtotal,^21^ if a small, non‐measurable tumor rim or lining was visible on the postoperative scans. Partial resection was defined by a solid residue in postoperative MR imaging, while (stereotactic, endoscopic, or microsurgical) biopsy left the tumor radiologically unchanged. If biopsy was followed by tumor resection during one hospital stay in a two‐staged approach, both interventions were counted as one, and the extent of resection was rated according to the definitive therapeutic surgery.

### 2.4 Neuroimaging

Central radiologic review for primary tumor location, extent of surgery, and long‐term tumor status was performed at the Reference Center for Neuroradiology of the German Society of Pediatric Oncology and Hematology (GPOH), University of Wuerzburg, Germany. Neuro‐imaging followed recommendations of the German pediatric brain tumor network (“HIT‐Netzwerk”)^22^ and published consensus by the European SIOP Brain Tumor Imaging Group.^21^

### 2.5 Statistical analysis

For categorical variables, absolute or relative frequencies are given. Continuous variables are described by median and range. The impact of the time interval (TI1‐5), age at diagnosis, major tumor site, sex, neurofibromatosis‐ (NF1‐) and tuberous sclerosis complex‐ (TSC‐) status on the pattern of initial surgery was analyzed with multivariable logistic regression. Multivariable model building is described in Data S1.

The German LGG studies had not included predefined neurosurgical questions with respect to the extent of resection. Therefore, all analyses were exploratory, and *p*‐values were considered as descriptive measures to detect and study meaningful effects.

## 3 RESULTS

### 3.1 Patient cohort

Between October 1st, 1996, and December 31st, 2018, 4317 patients had been registered prospectively into HIT‐LGG 1996 (*n* = 899), SIOP‐LGG 2004 (*n* = 1582), and the LGG registry (*n* = 1836). Patients with a date of diagnosis prior to 01.10.1996 (prior to HIT‐LGG 1996) were excluded (*n* = 47); thus, 4270 patients were eligible for analysis and grouped within the five time intervals. Successive cohorts did not differ in their epidemiologic data, which are summarized in Table 1. Patient numbers for the various analyses are detailed in Figure S1.

**TABLE 1:** Epidemiologic characteristics of the entire cohort (all patients: *n* = 4270) and of patients with at least 1 surgical intervention by time interval (TI) (*n* = 3440/4270).

	All patients *n* = 4270 (%)	Group TI1 *n* = 812 (%)	Group TI2 *n* = 634 (%)	Group TI3 n= 724 (%)	Group TI4 *n* = 631 (%)	Group TI5 *n* = 639 (%)
Median age (years, range)						
At diagnosis	7.57 (0.01–17.04)	7.37 (0.04–16.95)	8.16 (0.29–17.61)	8.62 (0.05–17.89)	8.70 (0.25–17.94)	8.33 (0.03–17.82)
At 1st surgery (*n* = 3440)	8.48 (0.03–20.96)	7.57 (0.04–17.48)	8.48 (0.29–20.96)	9.23 (0.20–17.95)	9.12 (0.25–18.99)	8.49 (0.03–17.82)
Age group (years)						
<1	211 (4.9%)	48 (5.9%)	30 (4.7%)	29 (4.0%)	22 (3.5%)	27 (4.2%)
1–4	1212 (28.4%)	238 (29.3%)	153 (24.1%)	164 (22.7%)	163 (25.8%)	165 (25.8%)
5–9	1290 (30.2%)	263 (32.4%)	199 (31.4%)	231 (31.9%)	168 (26.6%)	185 (29.0%)
10–15	1368 (32.1%)	245 (30.2%)	228 (36.0%)	256 (35.4%)	234 (37.1%)	223 (34.9%)
>16	189 (4.4%)	18 (2.2%)	24 (3.8%)	44 (6.1%)	44 (7.0%)	39 (6.1%)
Sex						
Male	2245 (52.6%)	432 (53.2%)	343 (54.1%)	386 (53.3%)	346 (54.8%)	338 (52.9%)
Female	2025 (47.4%)	380 (46.8%)	291 (45.9%)	338 (46.7%)	285 (45.2%)	301 (47.1%)
Neurofibromatosis type 1						
Yes	628 (14.7%)	41 (5.0%)	28 (4.4%)	34 (4.7%)	30 (4.8%)	27 (4.2%)
No	3642 (85.3%)	771 (95.0%)	606 (95.6%)	690 (95.3%)	601 (95.2%)	612 (95.8%)
Tuberous sclerosis complex						
Yes	56 (1.3%)	12 (1.5%)	11 (1.7%)	8 (1.1%)	3 (0.5%)	0
No	4214 (98.7%)	800 (98.5%)	623 (98.3%)	716 (98.9%)	628 (99.5%)	639 (100%)
Tumor localization						
Cerebral hemisphere	999 (23.3%)	177 (21.8%)	163 (25.7%)	214 (29.6%)	192 (30.4%)	187 (29.3%)
Temporal/other lobes	481/518	99/78	90/73	128/86	107/85	97/90
SML VPG/Di/Mes	1615 (37.8%) 940^a^/336/339	264 (32.5%) 136^a^/88/40	171 (27.0%) 69/57/45	173 (23.9%) 77/48/48	163 (25.8%) 73^a^/42/48	154 (24.1%) 69/47/38
Cerebellum	1224 (28.7%)	274 (33.7%)	228 (35.9%)	254 (35.1%)	220 (34.9%)	224 (35.1%)
Caudal brain stem	299 (7.0%)	67 (8.3%)	45 (7.1%)	56 (7.7%)	33 (5.2%)	53 (8.3%)
Spinal cord	129 (3.0%)	30 (3.7%)	26 (4.1%)	27 (3.7%)	21 (3.3%)	20 (3.1%)
Primary multifocal	4 (0.1%)	0	1 (0.2%)	0	2 (0.3%)	1 (0.2%)
Dissemination (primary and secondary)						
Yes	190 (4.5%)	42 (5.2%)	29 (4.6%)	42 (5.8%)	31 (4.9%)	38 (5.9%)
No	4074 (95.4%)	769 (94.7%)	605 (95.4%)	682 (94.2%)	597 (94.6%)	599 (93.7%)
Not documented	6 (0.1%)	1 (0.1%)	0	0	3 (0.5%)	2 (0.3%)
Extent of 1st resection						
Complete	1310 (30.7%)	319 (39.3%)	253 (39.9%)	292 (40.3%)	260 (41.2%)	186 (29.1%)
Subtotal	402 (9.4%)	163 (20.1%)	73 (11.5%)	72 (9.9%)	40 (6.3%)	54 (8.5%)
Partial	981 (23.0%)	154 (19.0%)	177 (27.9%)	213 (29.4%)	195 (30.9%)	242 (37.9%)
Biopsy^b^	710 (16.6%)	176 (21.7%)	129 (20.3%)	142 (19.6%)	124 (19.7%)	139 (21.8%)
Other^c^	37 (0.9%)	0	2 (0.3%)	5 (0.7%)	12 (1.9%)	18 (2.8%)
No resection	830 (19.4%)	n.a.	n.a.	n.a.	n.a.	n.a.
Timing of 1st surgery						
Within 28 days	2669 (62.5%)	658 (81.0%)	510 (80.4%)	544 (75.1%)	453 (71.8%)	504^d^ (78.9%)
From 29 to 90 days	296 (6.9%)	77 (9.5%)	45 (7.1%)	64 (8.8%)	62 (9.8%)	48 (7.5%)
From 91 to 365 days	235 (5.5%)	37 (4.6%)	41 (6.5%)	42 (5.8%)	55 (8.7%)	60 (9.4%)
After >1 year	240 (5.6%)	40 (4.9%)	38 (6.0%)	74 (10.2%)	61 (9.7%)	27 (4.2%)
No resection	830 (19.4%)	n.a.	n.a.	n.a.	n.a.	n.a.
Number of patients documented with >1 surgery						
	n.a.^e^	n.a.^e^	160 (25.2%)	177 (24.4%)	133 (21.1%)	110 (17.2%)
Non‐surgical therapy						
Yes	1254^f^ (29.4%)	287 (35.3%)	172 (27.1%)	177 (24.4%)	149 (23.6%)	139 (21.8%)
No	3016 (70.6%)	525 (64.7%)	462 (72.9%)	547 (75.6%)	482 (76.4%)	500 (78.2%)
Timing of non‐surgical therapy for those treated						
Prior to 1st surgery	n.a.	14 (4.9%)	17 (9.9%)	21 (11.9%)	20 (13.4%)	8 (5.8%)
After 1st surgery	n.a.	272 (94.8%)	151 (87.8%)	153 (86.4%)	129 (86.6%)	131 (94.2%)
Not documented^g^	n.a.	1 (0.3%)	4 (2.3%)	3 (1.7%)	0	0
Histology (main groups)						
PA^h^	2298 (53.8%)	629 (77.5%)	426 (67.2%)	457 (63.1%)	385 (61.0%)	401 (62.8%)
DG2^i^	227 (5.3%)	56 (6.9%)	55 (8.7%)	58 (8.0%)	31 (4.9%)	27 (4.2%)
Glioneuronal tumors^j^	581 (13.6%)	83 (10.2%)	104 (16.4%)	128 (17.7%)	136 (21.6%)	130 (20.3%)
Other LGG^k^	272 (6.4%)	36 (4.4%)	42 (6.6%)	63 (8.7%)	65 (10.3%)	66 (10.3%)
Other diagnoses^l^	62 (1.5%)	8 (1.0%)	7 (1.1%)	18 (2.5%)	14 (2.2%)	15 (2.3%)
No histology	830 (19.4%)	n.a.	n.a.	n.a.	n.a.	n.a.
Median observation time (years, range)						
Surviving patients at last follow‐up	8.18 (0.00–23.06)	16.76 (0.0–23.06)	12.10 (0.10–16.12)	8.7 (0.63–12.05)	5.38 (0.0–8.35)	2.03 (0.0–4.80)
Survival time for patients who died	3.69 (0.03–20.08)	–	–	–	–	–
Survival						
Yes	4112	741	603	701	622	632
No	158^m^	71	31	23	9	7
Tumor status at the end of the observation period (surviving patients)						
Complete remission	1569	411	306	354	280	210
Stable disease^n^	2275	291	268	323	298	324
Progressive disease	96	21	11	9	21	18
Not known/not documented	172	18	18	15	23	80

Abbreviations: DG2, diffuse glioma WHO grade II; Di, diencephalon; Mes, mesencephalon; n.a., not applicable; PA pilocytic astrocytoma; PLNTY, Polymorphous low‐grade neuroepithelial tumor of the young analogue WHO grade I; SML, supratentorial midline; VPG, visual pathway glioma.

^a^ Including 1 tumor without exact assignment within the SML.

^b^ Comprising open, stereotactic, and endoscopic biopsy.

^c^ Comprising surgery of metastases (*n* = 7), extent of resection not documented (*n* = 30).

^d^ Including 1 patient operated for hydrocephalus with an inconclusive biopsy, but with radiologic tumor diagnosis on the early postoperative MRI.

^e^ Subsequent interventions had not been systematically documented in TI1; second and further tumor surgeries were reported for 580 patients from TI2‐5 with declining frequency in the more recent time intervals paralleling shorter follow‐up periods.

^f^ With respect to the first non‐surgical treatment; *n* = 330 without any surgery, *n* = 80 prior to 1st surgery, *n* = 836 following 1st surgery, *n* = 8 date of start not documented (*n* = 7 everolimus, *n* = 1 vincristine/carboplatin); start of non‐surgical treatment: median 125 days after diagnosis (range: 1 day‐14.31 years) for 330 patients without surgery, median 151 days (range: 0 days‐13.92 years) for 836 patients following biopsy (*n* = 432), partial (*n* = 294), subtotal (*n* = 72) or complete resection (*n* = 28) (other interventions *n* = 10).

^g^ Date of start of therapy not documented for *n* = 8 patients (7 received everolimus, 1 received chemotherapy).

^h^ Including pilomyxoid astrocytoma in groups 2 (*n* = 6), 3 (*n* = 13), 4 (*n* = 8), and 5 (*n* = 7).

^i^ Comprising astrocytoma WHO grade II (*n* = 198), oligodendroglioma WHO grade II (*n* = 16), oligo‐astrocytoma WHO grade II (*n* = 13).

^j^ Comprising desmoplastic infantile ganglioglioma/astrocytoma (*n* = 32), ganglioglioma/PLNTY (*n* = 381), dysembryoplastic neuro‐epithelial tumor (*n* = 148), rosette forming glioneuronal tumor (*n* = 17), papillary glioneuronal tumor (*n* = 3).

^k^ Comprising subependymal giant cell astrocytoma WHO grade I (*n* = 47), angiocentric glioma WHO grade I (*n* = 17), LGG not otherwise specified (*n* = 78), pleomorphic xanthoastrocytoma WHO grade II (*n* = 49).

^l^ Comprising no tumor tissue in pathologic specimen (*n* = 56), not documented (*n* = 3), anaplastic astrocytoma WHO grade 3 in biopsy following 2, 6 and 12 years after clinical/radiological diagnosis of LGG (*n* = 3).

^m^ Causes of death: progression of primary tumor *n* = 64, not documented *n* = 28, malignant transformation *n* = 23, unrelated to tumor *n* = 13 (e.g., accident), treatment related *n* = 14 (non‐surgical treatment *n* = 8, surgical treatment *n* = 6), other neoplasia *n* = 8 (NF1‐associated *n* = 5, non‐NF1 *n* = 3), associated with shunt dysfunction *n* = 3, malignant primary at review *n* = 3, associated with epilepsy *n* = 2.

^n^ Including tumors responding to therapy.

Up to August 4th, 2020, tumors of 830 patients (19.4%) had been diagnosed radiologically, while at least one surgical intervention had been reported for 3440 patients (80.6%). Non‐surgical treatment was given to 1254 patients representing variable fractions from the main tumors locations (SML 52.8%, caudal brainstem 47.0%, spinal cord 32.5%, cerebral hemispheres 11.2%, cerebellum 6.5%).

### 3.2 Descriptive data over time

**FIGURE 1:**
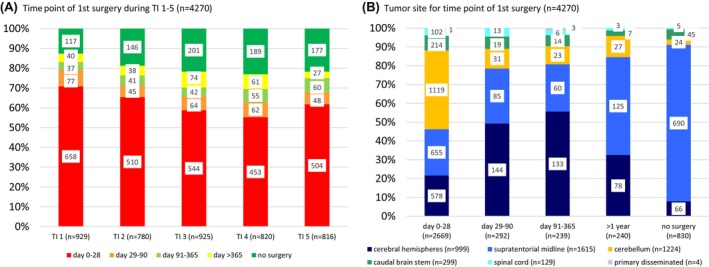
A: Time point of first intervention in relation to the date of diagnosis per TI, B: tumor sites per time point of first intervention.

The fraction of radiological LGG diagnoses increased over time (TI1 12.6%; TI5 21.7%). The majority of operated patients had their first intervention within 28 days (55.2%–70.8% among time intervals), while surgery was timed between days 29 and 90 in 5.8%–8.3% and 91 and 365 in 4.0%–7.4%. First surgery after observation of >1 year (range 1–14 years) was performed in a total of 240/3440 patients (7.0%) (Figure 1A).

About 18/3200 patients (0.6%) received chemotherapy (*n* = 17) or radiotherapy (*n* = 1) (15/18 SML tumor) prior to intervention within the first year after diagnosis (3200/3440). Non‐surgical treatment preceded delayed first interventions (>1 year) in 62/240 patients (25.8%). The fraction of patients with chemotherapy/radiotherapy after surgery declined, corresponding to shorter observation times (TI1: 33.5%; TI5: 20.5%).

**FIGURE 2:**
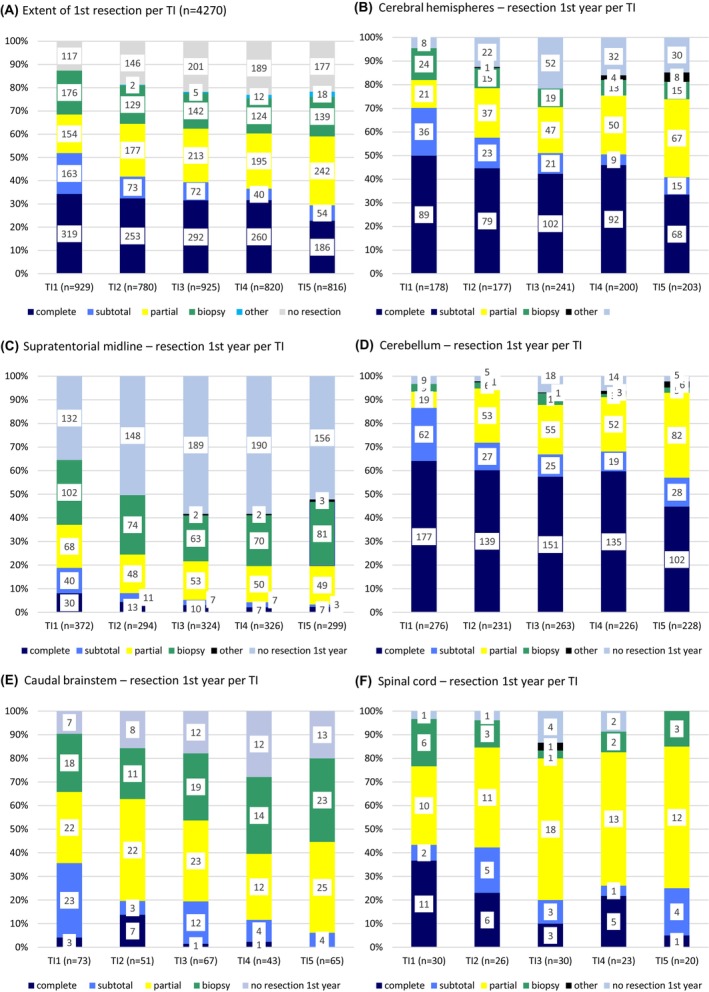
Extent of first resection for all and per tumor site. (A) Extent of first resection within the time intervals TI1‐5; (B–F) Extent of first resection for interventions in the first year following diagnosis for tumor site per time interval TI1‐5: B: cerebral hemispheres, C: supratentorial midline, D: cerebellum, E: caudal brainstem, F: spinal cord.

The extent of the first intervention showed a reduced fraction of complete/subtotal resections from TI1 to TI5, while the fraction of partial resections increased. The fraction of biopsies was similar during all TIs and made up for around 20%. (Figure 2A).

#### 3.2.1 Extent of first resection by interval between diagnosis and surgery (*n* = 3440)

Surgical interventions scheduled within the first 28 days (2669/3440) resulted in complete resection of 41.2%–42.5% of tumors in TI1‐4 and in 29.2% in TI5. The fraction of complete removals increased in TI4 for resections delayed beyond day 29. From TI1 to TI5, subtotal resections became infrequent, while the portion of partial resections increased. During all five study intervals, biopsy was performed in 15.3%–19.9% of tumors from day 1 to 28, yet up to 34.1% during later interventions within the first year. The majority of 240 patients with first surgery >1 year had biopsies (40.0%) or partial resections (25.8%), still 29.6% tumors were completely resected (Figure S2A–D and Table S1).

#### 3.2.2 Relation of the interval between diagnosis and first surgery and tumor site (*n* = 3440)

The majority of tumors with resection between days 1 and 28 were located in the cerebellum, the SML, and the cerebral hemispheres. At later time points within the first year, the fraction of hemispheric tumors increased, to a lesser extent for SML sites as well, while interventions for cerebellar LGG made up less than 10% (Figure 1B). SML LGG made up for 52.1% of 240 interventions beyond the first year (Table S2), while 23.3% of those were NF1‐associated.

Among early interventions, there was a small shift towards more cerebral (TI1: 18.2%; TI2‐5: 21.2%–24.6%) and cerebellar (TI1: 39.1%; TI2‐5: 42.0%–44.6%) tumors over time, while the portion of SML LGG with up‐front surgery declined (TI1: 30.1%; TI2‐5: 20.8%–24.1%).

#### 3.2.3 Extent of first resection by primary tumor site (*n* = 3440)

The pattern of the initial surgical procedure over time was analyzed for the first tumor resection within the first year after diagnosis (3200/3440) (Table 2 and Figure 2B–F).

**TABLE 2:** Extent of resection for tumor site by time interval (TI) (*n* = 4270).

	Group TI1 *n* = 929	Group TI2 *n* = 780	Group TI3 *n* = 925	Group TI4 *n* = 820	Group TI5 *n* = 816
Cerebral hemispheres	*n* = 178	*n* = 177	*n* = 241	*n* = 200	*n* = 203
Surgery within 1st year	170	155	189	168	173
Complete resection	89	79	102	92	68
Subtotal resection	36	23	21	9	15
Partial resection	21	37	47	50	67
Biopsy	24	15	19	13	15
Other^a^	0	1	0	4	8
Surgery delayed >1 year	7	8	25	24	14
Follow‐up <1 year	0	0	0	0	2
Radiological diagnosis	1	14	27	8	14
Temporal lobe	*n* = 87	*n* = 75	*n* = 118	*n* = 102	*n* = 99
Surgery within 1st year	84	66	94	89	85
Complete resection	40	33	51	47	29
Subtotal resection	17	9	10	5	6
Partial resection	14	17	25	27	38
Biopsy	13	7	8	7	5
Other^a^	0	0	0	3	7
Surgery delayed >1 year	2	4	14	10	8
Radiological diagnosis	1	5	10	3	6
SML	*n* = 372	*n* = 294	*n* = 324	*n* = 326	*n* = 299
Surgery within 1st year	240	146	135	136	143
Complete resection	30^b^	13^b^	10^b^	7^b^	7^b^
Subtotal resection	40	11	7	7	3
Partial resection	68	48	53	50	49
Biopsy	102	74	63	70	81
Open/ster/endo	45/56/1	22/47/5	18/34/11	20/36/14	24/28/29
Other^a^	0	0	2	2	3
Surgery delayed >1 year	24	25	38	27	11
Follow‐up <1 year	4	1	0	4	22
Radiological diagnosis	104	122	151	159	123
Cerebellum	*n* = 276	*n* = 231	*n* = 263	*n* = 226	*n* = 228
Surgery within 1st year	267	226	245	212	223
Complete resection	177	139	151	135	102
Subtotal resection	62	27	25	19	28
Partial resection	19	53	55	52	82
Biopsy	9	6	13	3	5
Other^a^	0	1	1	3	6
Surgery delayed >1 year	7	2	9	8	1
Follow‐up <1 year	0	0	0	0	1
Radiological diagnosis	2	3	9	6	3
Caudal brain stem	*n* = 73	*n* = 51	*n* = 67	*n* = 43	*n* = 65
Surgery within 1st year	66	43	55	31	52
Complete resection	3	7	1	1	0
Subtotal resection	23	3	12	4	4
Partial resection	22	22	23	12	25
Biopsy	18	11	19	14	23
Other^a^	0	0	0	0	0
Surgery delayed >1 year	1	2	1	2	1
Follow‐up <1 year	0	0	1	0	4
Radiological diagnosis	6	6	10	10	8
Spinal cord	*n* = 30	*n* = 26	*n* = 30	*n* = 23	*n* = 20
Surgery within 1st year	29	25	26	21	20
Complete resection	11	6	3	5	1
Subtotal resection	2	5	3	1	4
Partial resection	10	11	18	13	12
Biopsy	6	3	1	2	3
Other^a^	0	0	1	0	0
Surgery delayed >1 year	1	1	1	0	0
Follow‐up <1 year	0	0	0	0	0
Radiological diagnosis	0	0	3	2	0
Primary dissemination	*n* = 0	*n* = 1	*n* = 0	*n* = 2	*n* = 1
Type of surgery		biopsy		other^a^	biopsy

*Note*: Patients are grouped for extent of resection of 1st surgery within the 1st year after diagnosis (*n* = 3200). Number of patients given for delayed surgery >1 year (*n* = 240), for observation without surgery but follow‐up of <1 year^c^ (*n* = 39), and for radiological diagnosis without surgery and follow‐up of >1 year (*n* = 791).

Abbreviations: open/ster/endo, type of biopsy – open, stereotactic, endoscopic; SML, supratentorial midline.

^a^ Other interventions: surgery of metastases only, extent of surgery not documented.

^b^ Complete resection of tumors in the anterior optic nerve in case of visual loss 4/30 (TI1), 5/13 (TI2), 2/10 (ZI3), 3/7 (TI4), and 3/7 (TI5).

^c^ Follow‐up with an observation time of <1 year: patients died *n* = 6, lost to follow‐up *n* = 33.

Resection of cerebral hemispheric and cerebellar tumors was mostly complete/subtotal with a declining relative fraction from TI1 (73.5% and 89.5%) to TI5 (48.0% and 58.3%), but an intermittent rise for hemispheric LGG in TI4. In return, the relative fraction of partially resected LGG increased, while biopsy concerned a largely constant, small portion. At all times, temporal LGG were resected less completely compared to the other cerebral regions.

Within the first year, the fraction of completely/subtotally resected SML tumors showed a decline from TI1 (29.2%) to TI2 (16.4%) to below 6% thereafter, while a rising portion was biopsied (T1: 42.5%; T5: 56.6%). Over time, the stereotactic technique was less often used for biopsies, while more endoscopic interventions were performed (Figure S3).

Comparable relative changes occurred in the brainstem (complete/subtotal resections: TI139.4%, TI4‐5 < 10%; more partial resections) and in the spinal cord (complete/subtotal resections: TI1 44.8%, TI5 7.7%; partial resections: TI1 34.5%, TI5 60.0%).

#### 3.2.4 Extent of resection for NF1‐associated LGG (*n* = 628)

Most NF1‐associated LGG were diagnosed radiologically (74.5%; 468/628), while a quarter (25.5%; 160/628) had histologic confirmation (Table S3).

NF1‐associated tumors resected within the first year or with follow‐up of at least 1 year (554/628) were mainly located in the SML (84.7%) and caudal brainstem (7.2%). Surgery was performed on 104/554 tumors. The extent of resection declined for complete/subtotal resection (TI1 31.0%; TI5 4.5%) and increased markedly for biopsies (TI1 34.5%; TI5 77.3%). The portion of any intervention increased for brainstem lesions (TI1: 6.9%; TI3: 21.1%; TI5: 31.8%) and declined in the SML (TI1: 65.5%; TI3: 42%; TI5: 50%).

#### 3.2.5 Extent of resection by patient age group (*n* = 3440)

**FIGURE 3:**
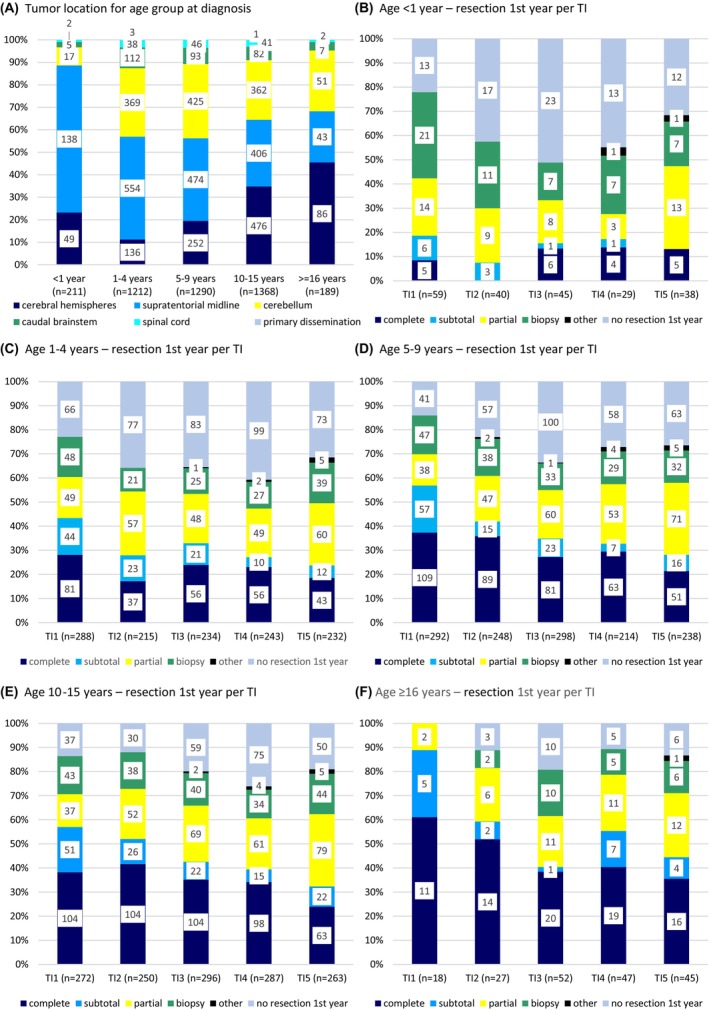
Tumor site and extent of first resection for age. (A) Tumor location for age group at diagnosis; (B–F) Extent of first resection for interventions in the first year following diagnosis for age group at diagnosis per time interval TI1‐5: B: <1 year, C: 1–4 years, D: 5–9 years, E: 10–15 years, F: ≥16 years.

Main tumor localization shifted with age, showing a majority of SML‐LGG in infants and an increasing portion of tumors in the cerebral hemispheres in older children (Figure 3A).

The LGG in infants was diagnosed radiologically in more than a quarter (26.1%) but less often in older patients (≥16 years: 10.6%). If operated, infant LGGs were less often resected completely (11.4% vs. 42.9% in patients ≥16 years), while they were biopsied more often (30.8% vs. 13.2%–16.6% in the other age groups), with no major differences for subtotal and partial resections.

For resections within the first year following diagnosis, the fraction of complete/subtotal resections declined with an increase of partial resections in all age groups over time (Figure 3B–F and Table S4A–E).

#### 3.2.6 Extent of resection by histology for first interventions within the first year (*n* = 3200)

It is acknowledged that during the 22‐year registration period, the histologic classification of LGGs was subject to multiple revisions including the definition of new entities.^23^

Pilocytic astrocytoma (PA) remained the largest group of diagnoses during all time intervals (TI1: 77.5%; TI5: 62.8%). Most PA were resected completely, the fraction of subtotal resections declined and rose for partial resections (18.7% to 42.2%, respectively). Biopsies were performed in 12.5%–19.8%.

Most diffuse glioma WHO grade II were biopsied (36.5%–46.2%) or resected partially (increasing from 16.6 to 30.8%). A quarter of lesions could be resected completely (26.9%–23.1%), while subtotal resections became rare.

The group of glioneuronal LGG grew throughout the recruitment time from 10.2% to 20.3%. The extent of resection within the first year changed with less complete (53.2% to 34.2%), and subtotal (16.5% to 6.7%), more partial resections (20.3% to 40.0%), and 9.6%–13.3% biopsies.

The umbrella term of “other LGG” included SEGA, pleomorphic xanthoastrocytoma WHO grade II, and LGG without further specification. Their fraction grew from 4.4% to 10.3%, but subgroups remained too small for further analysis. The extent of resection followed the general trends (Table S5).

#### 3.2.7 Outcome data

Within the observation time, 158/4270 patients died (3.7%; in median after 3.7 years); a large portion succumbed to progression of their LGG (64/158), though the cause of death had not always been recorded.

At the end of the observation time (median 8.7 years), 38.2% of 4112 surviving patients were in complete remission, 55.3% had stable disease, and 2.3% of tumors were progressive.

Among 666/4270 patients (15.6%) from TI2‐5 with incompletely resected tumors and no additional treatment during follow‐up, 92 patients attained radiological complete remission, 547 had stable disease, 18 tumors were progressive, and 9 patients had died at the time of the last information (median follow‐up 5.9 years). The majority of 914/4270 patients (21.4%) from TI2‐5 with complete initial resection and no further treatment remained in complete remission (Figure S4).

### 3.3 Inferential statistical results

The pattern of the initial surgical procedure over time was analyzed for the first tumor resection status within the first year after diagnosis. Analyses included all patients with at least one surgery (*n* = 3440) as well as those patients with an observation time of at least 1 year without tumor surgery (791/830) (in total 4231/4270). For these analyses, the resection status of 240/3440 patients with surgery beyond 1 year was censored as not having surgery within the first year, while 39/830 radiologically diagnosed pLGG patients with an observation time of <1 year were excluded from analyses as missing values (*n* = 6 died, *n* = 33 without adequate follow‐up information, 22/33 in TI5).

Time groups differed for median age at diagnosis (lower in TI1) and the fraction of patients within age subgroups, while epidemiologic data were comparable for sex ratio, NF1‐status, tumor sites (though growing number of older patients with hemispheric tumors in later time intervals), and dissemination (Table S6).

Binary logistic main effects models (Table 3) revealed steadily declining odds for complete and subtotal resection over time, arriving at an odds ratio of 0.405 and 0.333, respectively, comparing time interval TI5 to TI1. The odds for complete resection were higher in the cerebral hemispheres, the cerebellum, or the spinal cord as compared to the SML or the brainstem. The odds for partial resection rose in the course of time (odds ratio of 2.658 for TI5 vs. TI1) and were notably increased in the brainstem and spinal cord. The odds for biopsy showed no clear change over time. Increasing age at diagnosis only augmented the odds for complete resection. NF1‐ and TSC‐negative patients had higher odds for any extent of resection; sex was non‐influential.

**TABLE 3:** Results of binary logistic regression analysis examining the extent of neurosurgical resection.

Parameter	Odds‐Ratio^a^	(95%‐CI)		*p*‐value^b^
Complete resection				
Time Interval				<0.001
2 versus 1	0.779	0.607	0.999	0.049
3 versus 1	0.626	0.493	0.795	<0.001
4 versus 1	0.692	0.540	0.887	0.004
5 versus 1	0.405	0.314	0.523	<0.001
Age at diagnosis				<0.001
1–4 years versus <1year	2.159	1.268	3.674	0.005
5–9 years versus <1year	2.792	1.653	4.714	<0.001
10–15 years versus <1year	3.149	1.874	5.293	<0.001
≥16 years versus <1year	3.894	2.134	7.108	<0.001
Localization				<0.001
Hemisphere versus SML	12.925	9.717	17.193	<0.001
Cerebellum versus SML	23.715	17.941	31.348	<0.001
Caudal BS versus SML	0.777	0.413	1.462	0.434
Spinal cord versus SML	4.323	2.613	7.152	<0.001
NF 1: no versus yes	3.921	2.369	6.490	<0.001
TSC: no versus yes	4.063	2.062	8.006	<0.001
Sex: female versus male	—	—	—	0.918
b Subtotal resection				
Time Interval				<0.001
2 versus 1	0.445	0.328	0.604	<0.001
3 versus 1	0.361	0.266	0.490	<0.001
4 versus 1	0.245	0.170	0.353	<0.001
5 versus 1	0.333	0.239	0.463	<0.001
Age at diagnosis	—	—	—	0.442
Localization				<0.001
Hemisphere versus SML	2.129	1.535	2.953	<0.001
Cerebellum versus SML	2.581	1.904	3.499	<0.001
Caudal BS versus SML	3.447	2.289	5.191	<0.001
Spinal cord versus SML	2.262	1.237	4.137	0.008
NF 1: no versus yes	6.325	2.930	13.656	<0.001
TSC: no versus yes	5.244	1.703	16.151	<0.001
Sex: female versus male	—	—	—	0.918
c Partial resection				
Time Interval				<0.001
2 versus 1	1.684	1.306	2.171	<0.001
3 versus 1	1.638	1.280	2.094	<0.001
4 versus 1	1.748	1.359	2.250	<0.001
5 versus 1	2.658	2.084	3.392	<0.001
Age at diagnosis	—	—	—	0.191
Localization				<0.001
Hemisphere versus SML	0.932	0.758	1.146	0.504
Cerebellum versus SML	0.901	0.739	1.098	0.300
Caudal BS versus SML	2.257	1.699	2.998	<0.001
Spinal cord versus SML	3.729	2.546	5.462	<0.001
NF 1: no versus yes	7.671	5.115	11.506	<0.001
TSC: no versus yes	5.586	2.320	13.450	<0.001
Sex: female versus male	—	—	—	0.918
d Biopsy				
Time Interval				0.057
2 versus 1	0.848	0.638	1.126	0.253
3 versus 1	0.770	0.583	1.016	0.065
4 versus 1	0.741	0.556	0.988	0.041
5 versus 1	1.063	0.807	1.401	0.662
Age at diagnosis	—	—	—	0.795
Localization				<0.001
Hemisphere versus SML	0.203	0.157	0.262	<0.001
Cerebellum versus SML	0.064	0.045	0.091	<0.001
Caudal BS versus SML	0.991	0.745	1.317	0.950
Spinal cord versus SML	0.291	0.167	0.507	<0.001
NF 1: no versus yes	5.115	3.719	7.034	<0.001
TSC: no versus yes	8.954	2.048	39.150	<0.001
Sex: female versus male	—	—	—	0.918

Abbreviations: BS, brainstem; NF1, neurofibromatosis type 1; SML, supratentorial midline; TSC, tuberous sclerosis complex.

^a^ Odds‐Ratio of selected variables in the final model. Odds calculated as P/(1−P) with P = “Probability of the currently examined extent of resection.”

^b^ *p*‐Value of Wald test in final model/step of removal for selected/non‐selected variables.

A noticeable interaction with the time interval (*p* ≤ 0.05) was found for the major tumor sites only. For this reason, additional binary logistic regression models were fitted in the subgroups defined by the major tumor sites. This analysis for the major tumor sites (Table S7) confirmed the steady decline of odds for complete and subtotal resection over time in the SML, cerebral hemispheres, and cerebellum.

Age >1 year at diagnosis increased the odds for complete resection in the cerebellum, while only the age groups 10–15 and ≥16 years with their higher portion of hemispheric LGG, especially in the temporal lobe, had higher odds for complete resection in the cerebral hemispheres. Over time, the odds for partial resection rose in the hemispheres and cerebellum. In the SML, partial resections were more probable just within the age group 1–4 years at diagnosis. NF1‐negative patients had higher odds for any extent of resection in the SML. Sex was non‐influential for any analysis.

The decreasing likelihood over time (from TI1 to TI5) of complete/subtotal versus partial resection/biopsy in the SML, in the cerebral hemispheres, and in the cerebellum was corroborated by multinominal logistic regression analysis. Likewise, higher age at diagnosis increased the probability of complete/subtotal resection of hemispheric and SML LGG, with no impact of the NF1‐status at the SML (Table S8).

The decrease of extent of first neurosurgical intervention, however, did not compromise long‐term outcome. In fact, 5‐year OS improved from 95.9% in TI1 to 98.6% in TI5 (*p* < 0.001, log‐rank test).

## 4 DISCUSSION

Our data document the decrease of the extent of the first neurosurgical intervention without compromising the long‐term outcome of pediatric low‐grade glioma in Germany between 1996 and 2018, though technical advances that would support neurosurgical resection were introduced at the same time. The odds for having a complete resection at first operation within the first year after diagnosis declined by a factor 2.5 over the recruitment period. Binary logistic regression analysis confirmed the presence of these temporal trends beyond tumor site and age at diagnosis as major influential factors for the probability of a certain extent of resection. For this analysis, we included data on 4270 pLGG patients from three successive prospective and population‐based LGG studies with consistent recruitment criteria.^1^, ^7^, ^8^ We are not aware of a comparable publication analyzing the surgical resection patterns of a large pLGG cohort over more than 2 decades.

### 4.1 Patient cohort

Epidemiologic data of our cohort have been detailed previously^1^; they conformed to other international series with the exception of variable age limits for inclusion.^5^, ^6^, ^24^ Age grouping varied between cohorts as well, but usually allowed analyzing infant data separately.^6^, ^25^, ^26^, ^27^

Due to the joint analysis of successive studies, median observation time differed greatly between the cohorts of the five defined time intervals. Since nearly all patients had been followed for at least 1 year and 93% of all documented first surgical interventions were performed within the first year following diagnosis, we focused on the intervention patterns within the first year. Delayed first interventions (beyond 365 days) spread up to 14 years after diagnosis and reflected very individual courses.

### 4.2 Overall change of resection patterns over time

In the early 1990s, maximal aggressive surgery to guarantee high rates of progression‐free survival had been claimed for pLGG and for most CNS sites, even for SML lesions.^28^, ^29^, ^30^, ^31^ Gradually, realization of surgically induced late effects^4^, ^9^, ^10^, ^17^, ^32^, ^33^ and neurocognitive impairments after radiotherapy of tumor remnants,^34^, ^35^ as well as the advent of chemotherapy^7^, ^12^, ^15^, ^26^, ^27^ started a process of rethinking of treatment approaches.^11^, ^17^, ^32^, ^36^, ^37^ In parallel, the understanding grew that monodisciplinary decisions should be substituted for a state‐of‐the‐art multidisciplinary team (MDT) approach.^6^, ^16^, ^17^, ^36^, ^37^

International discussion questioned the benefit of radical surgical primarily for SML tumors, but resulting treatment recommendations were expanded to other sites. In our cohort, maximum tumor volume resection as first neurosurgical intervention dominated up to the year 2000, including a large fraction of subtotal resections. Starting from the early 2000s, the extent of first resection declined for SML LGG, while a marked reduction of the extent of resection occurred since the 2010s for tumors of the cerebral hemispheres and cerebellum. Over time, we observed more patients without any surgical intervention, more patients who had just an initial biopsy, and more patients with only partial resections. At the same time, the option to reach complete resection at second interventions became feasible, allowing to apply a safer strategy to preserve functional outcome in a low‐grade tumor with long life expectancy.^11^ However, timing and frequency of repeated resections reflect the length of the observation time and cannot yet be judged in our cohort for the last years.

### 4.3 Change of resections patterns for tumor sites

Discussions on the general role of the neurosurgeon for SML tumors^9^, ^30^ acknowledged an often extensive involvement of critical structures precluding complete removal, especially in NF1‐associated optic pathway gliomas (OPG). International consensus finally advocated not to attempt primary resection of chiasmatic‐hypothalamic gliomas and tumors of comparable sites; rather, tumor‐associated symptoms should be controlled by volume reduction, particularly if chemotherapy had failed or radiotherapy was not an option.^4^, ^11^, ^17^, ^32^ This change in attitude was mirrored in our cohort by a significant early decline of volume‐reducing resections, nearly abandoning complete/subtotal interventions during later time intervals in this subgroup of patients. Improved imaging allowed diagnosing more SML LGGs radiologically, a possible explanation for the early decline in the frequency of biopsies. Still, tissue diagnosis had been recommended for patients with atypical presentation of OPG all through,^16^, ^17^, ^32^ and was requested from the advent of molecular characterization and targeted therapy, even for unresectable (radiologically diagnosed) SML lesions.^37^, ^38^, ^39^, ^40^ Correspondingly, the number of biopsies rose in the mid‐2010s, at the transition from TI4 to TI5. Over time, the portion of endoscopic biopsies increased over the stereotactic approach, though most interventions in the optic pathways remained open biopsies when tumors had no ventricular proximity or there was a risk for bleeding. Yet, it has clearly been reported that postoperative complication rates seem lower for stereotactic and endoscopic interventions compared to open biopsies.^10^, ^32^, ^41^

The trend for less invasive interventions as well as the growing quest for adequate biopsies to obtain molecular genetic information^37^, ^42^ were mirrored in the NF1‐cohort as well.

Though surgery for SML‐LGG should be delayed as long as possible,^17^, ^32^ our data indicate that most patients had their first intervention within the first 3 months. Still, SML‐LGG represented more than half of all tumors with interventions >1 year after diagnosis; most were just biopsied.

While a larger percentage of patients had volume‐reducing surgery in the brainstem and spinal cord as compared to the SML, the extent of first resection declined at both sites.^43^, ^44^ A widely constant portion of brainstem tumors were just resected partially, after dorsally exophytic tumors had even been judged “amenable for subtotal resection in the 1990s”.^45^ As Histone3‐K27M‐mutated diffuse glioma WHO grade II had been found in these cohorts,^43^ the portion of biopsies grew in later time intervals. Nearly all spinal tumors had histologic confirmation with less complete/subtotal and more partial resections over time. The necessity for multiple interventions has been reported, while the majority of spinal cord LGG can be treated successfully with surgery alone.^11^, ^44^

Though the balance of radicality and post‐surgical morbidity differs for the cerebral hemispheres and the cerebellum if compared to midline structures,^11^ there are significant risks for long‐term, post‐surgical sequelae, e.g., the cerebellar mutism syndrome^46^ or neurocognitive impairments.^33^, ^47^, ^48^ In the past, event‐free survival remained higher for both sites following complete versus. incomplete resection, but overall survival did not differ relevantly.^5^, ^6^, ^7^, ^11^ Following the general recommendation for resection of amenable tumors at these sites,^5^, ^11^, ^16^, ^49^ >80% of all interventions in the cerebral hemispheres and >90% in the cerebellum concerned volume‐reducing surgery in our cohort, with more extensive resections in the cerebellum at all times. Still, a significant shift was observed with a relative reduction of complete/subtotal resections and a parallel increase of partial resections. Especially, the portion of subtotal resections diminished in the cerebral hemispheres, having been a frequent surgical result in the 1990s.^29^, ^50^ The additional impact of better radiological delineation of postoperative residues cannot be excluded.^21^ An intermittent increment of complete resections in the cerebellum and the temporal lobe in TI4 could be explained by backdated registration of patients treated “surgically only,”^1^ especially for patients receiving lesionectomy for epilepsy caused by ganglioglioma.

In total, interventions became less complete over time but not less frequent. Furthermore, almost 20% of our patients (with LGG of all sites) were in complete remission or stable disease at last follow‐up despite having had incomplete initial surgery without subsequent treatment. The contribution of specific mutations to postsurgical growth impairment, cellular senescence, or any other mechanism is as yet unsettled.^51^, ^52^

Corresponding to the frequent association of posterior fossa lesions with symptoms of increased intracranial pressure, cerebellar LGG underwent surgery mostly “at diagnosis,” while cerebral hemispheric LGG allowed for “more delayed” interventions. In parallel to less invasive procedures, such variable timing of interventions was most likely the result of detailed discussions in the MDTs.

### 4.4 Impact of histology

Analyses concerning the extent of first resection in relation to tumor histology over time reflect the trend of less complete first interventions. Yet, the distribution of the extent of resection for histological subgroups parallels their distribution within CNS sites. Thus, tumor site rather than histology determined the extent of surgical intervention, though some histologies, for example diffuse glioma WHO grade II, may be less resectable at certain sites.^49^ Even if diagnostic imaging revealed details of tissue composition, no current recommendation relates histology to a specific surgical approach.^16^

### 4.5 Impact of age

For patients with first surgery within 1 year of diagnosis or 1 year of observation without surgery, median age was 5.89 years for SML LGG and 11.28 years for LGG of the cerebral hemispheres. Thus, the impact of age upon the extent of tumor resection seems to be mainly a consequence of the age‐related shift in tumor location. The relative probability of complete resection increased with age, particularly as more LGG were located in the cerebral hemispheres and the cerebellum.^5^, ^6^ In addition, only few adolescents remained observed without surgery within our cohort. Nevertheless, the decrease of the extent of surgery over time could be traced even in this age group, while it was most clearly discernable in the 5–9 and 10–15 year age groups. For infants and the age group 1–4 years at diagnosis, the decrease of the extent of resection with a parallel trend for more radiologic diagnoses could only be seen in early time intervals (TI1‐3). During later intervals, their portion of histologically verified LGG increased by more biopsies and partial resections.

### 4.6 Non‐surgical therapy

The extent of surgical resection remains the pivotal point for indication and timing of post‐surgical therapy in almost all treatment algorithms since the 1990s.^6^, ^12^, ^13^, ^14^, ^15^, ^16^, ^26^, ^27^ Accordingly, the portion of patients receiving non‐surgical therapy in our cohort corresponded inversely to the extent of surgical volume reduction at the various LGG sites. The advent of everolimus in later TIs reduced recruitment of TSC patients, in whom surgical intervention became rare but who account for a small number of patients only. Due to small numbers, the impact of prior non‐surgical treatment upon surgery could not be judged.

## 5 LIMITATIONS

Despite the large, population‐based cohort, interpretation of our data and results is limited since German LGG studies focused on primary non‐surgical treatment; all other aspects, including surgery, were prospectively documented, but with a restricted data set.

Assessment of neurocognitive development was recommended within all German cooperative multicenter brain tumor studies only since the 2010s, but sample size of pLGG patients remained small^33^ precluding inclusion in the analysis of the extent of surgical resection over time.

Patient observation time relevantly differed between the three successive studies, limiting analysis to neurosurgical interventions during the first year following diagnosis.

Documentation of preoperatively defined surgical goals, intraoperative findings, and decisions during surgical intervention or of post‐surgical change in neurological condition and its course was not common practice during the first LGG study. Due to limited resources, such routine documentation was not established throughout the subsequent years.

Unfortunately, we currently lack molecular‐pathologic findings for the majority of tumors. The degree of infiltration and the probability of complete resection may be related to molecular subtypes. Molecular‐histologic characterization has been integrated into current German brain tumor studies^2^, ^53^ allowing it to be considered for all relevant future study questions.

## 6 CONCLUSIONS

Our results document a relevant change of the surgical resection pattern over time, whose most striking fact is a non‐random, declining portion of complete/subtotal resections. This trend does not relate to changes in patients' characteristics or anatomical conditions. In contrast, it can be correlated to the evolution of comprehensive treatment algorithms that assign indications and limitations to all therapeutic modalities respecting age, tumor site and size, and NF1‐status. Such seemingly less favorable results of the first surgical intervention may in fact be advantageous for long‐term neurocognitive outcome and quality of life, at least in certain age groups or tumor locations, while they did not compromise long‐term radiological outcome and survival.

The intended alignment to contemporary treatment algorithms conveys accepted surgical guidelines.^16^ The treatment plan must be discussed and agreed upon through the MDT before surgery occurs, including the reasons for surgery and the planned extent of resection tailored to the individual needs of each child. Surgery should be performed by a (pediatric) neurosurgeon with regular experience in the care of children with brain and spinal cord tumors and by techniques and approaches that minimize the risk of permanent postoperative deficits. Second‐look surgery should be considered by the MDT if (recurrent or progressive) tumor residues that warrant treatment can be safely removed.^11^

Although contemporary studies on pLGG continue to focus on (mostly targeted) non‐surgical therapies for non‐resected/non‐resectable tumors,^25^, ^37^, ^38^, ^39^ surgical aspects of the multimodal treatment strategy merit further investigation. Comprehensive documentation of the individual proceedings should include preoperative patient conditions, discussion and planning in the MDT (including the goal of surgery and possible second interventions), accurate surgical and anesthetic reports, and postoperative patient condition and course.^10^ Linking surgical documentation, post‐surgical and medical/ophthalmologic morbidity, and long‐term neurocognitive development might also allow to better delineate the respective roles for patient outcome and support further development of treatment algorithms.^33^ It is of key importance to understand how planning, performance, or tumor characteristics impact upon achieving surgical goals.

## AUTHOR CONTRIBUTIONS

**Tibor Kelety:** Conceptualization (equal); data curation (equal); formal analysis (equal); methodology (equal); writing – original draft (equal); writing – review and editing (equal). **Ulrich‐Wilhelm Thomale:** Conceptualization (equal); data curation (equal); formal analysis (equal); methodology (equal); writing – original draft (equal); writing – review and editing (equal). **Daniela Kandels:** Data curation (equal); writing – original draft (supporting); writing – review and editing (supporting). **Martin U. Schuhmann:** Writing – original draft (supporting); writing – review and editing (supporting). **Ahmed El Damaty:** Writing – original draft (supporting); writing – review and editing (supporting). **Jürgen Krauss:** Writing – original draft (supporting); writing – review and editing (supporting). **Michael C. Frühwald:** Writing – original draft (supporting); writing – review and editing (supporting). **Pablo Hernáiz Driever:** Writing – original draft (supporting); writing – review and editing (supporting). **Olaf Witt:** Writing – original draft (supporting); writing – review and editing (supporting). **Brigitte Bison:** Writing – original draft (supporting); writing – review and editing (supporting). **Monika Warmuth‐Metz:** Writing – original draft (supporting); writing – review and editing (supporting). **Torsten Pietsch:** Writing – original draft (supporting); writing – review and editing (supporting). **René Schmidt:** Conceptualization (supporting); formal analysis (lead); methodology (equal); writing – original draft (equal); writing – review and editing (supporting). **Astrid K. Gnekow:** Conceptualization (lead); data curation (lead); formal analysis (supporting); funding acquisition (lead); investigation (lead); methodology (supporting); project administration (lead); resources (lead); supervision (lead); validation (lead); writing – original draft (lead); writing – review and editing (lead).

## CONFLICT OF INTEREST STATEMENT

All authors declared no conflicts of interest.

## ETHICS STATEMENT

Informed consent was obtained from patients, parents, and/or guardians. The HIT‐LGG 1996 study was approved by local and central ethic boards. The SIOP‐LGG 2004 study and the LGG‐registry adhered to the Declaration of Helsinki in its revised version (Edinburgh, Scotland, 2000), the WHO and European Community rules of “Good Clinical Practice” (effective January 17, 1997). The protocols of the SIOP‐LGG 2004 study and the LGG‐registry were ethically approved by the Ethical committee of the Ludwig Maximilian's University of Munich, Germany (No. 179‐08 and No. 136‐12).

## Supporting information


Data S1.

## Data Availability

The data sets for the current study are available from the corresponding author on reasonable request.
