# Supplementary material for: Adaption of neurosurgical resection patterns for pediatric low‐grade glioma spanning two decades—Report from the German LGG‐studies 1996–2018
Source: Cancer Med. 2024 Jun 24;13(12):e7417. doi: 10.1002/cam4.7417 (PMC11194681; doi:10.1002/cam4.7417)

# **Adaption of neurosurgical resection patterns for pediatric low-grade glioma during two decades – report from the German LGG-studies 1996-2018**

Tibor Kelety^1^, Ulrich-Wilhelm Thomale^2^, Daniela Kandels^1^, Martin U Schuhmann^3^, Ahmed ElDamaty^4^, Juergen Krauss^5^, Michael Fruehwald^1^, Pablo Hernáiz Driever^6^, Olaf Witt^7^, Brigitte Bison^8^, Torsten Pietsch^9^, René Schmidt^10^, Astrid K. Gnekow^1^

**Supplementary material:**

- **Multivariable model building**
- **Supplementary table 1:**

Extent of resection for interval between diagnosis and 1^st^ surgery per time interval (TI) (n=3440)

- **Supplementary table 2:**

Tumor localization for interval between diagnosis and 1^st^ surgery per time interval (TI) (n=3440)

- **Supplementary table 3:**

Extent of 1^st^ resection for NF1-associated LGG and tumor site per time interval (TI) (n=628)

- **Supplementary table 4:**

Extent of 1^st^ resection for age group at diagnosis and tumor site per time interval (TI) (n=4270)

- 1. age <1 year
  2. age 1-4 years
  3. age 5-9 years
  4. age 10-15 years
  5. age ≥16 years
- **Supplementary table 5:**

Extent of 1^st^ resection for main histologic subgroups per time interval (TI) (n=4270)

- **Supplementary table 6**

Univariable analysis of the distribution of epidemiologic data per time interval (TI) (n=4231; n=39 patients without known surgical intervention excluded for follow-up time <1 year)

1. median patient age at diagnosis
2. age groups
3. sex (male/female)
4. NF1 status
5. TSC status
6. tumor localization
7. tumor dissemination

- **Supplementary table 7:**

Results of the binary logistic regression analysis examining the extent of neurosurgical resection for tumor site

1. Supratentorial midline (n=1584)
2. Cerebral hemispheres (n=997)
3. Cerebellum (n=1223)

- **Supplementary table 8**

Results of multi-nominal logistic regression analysis examining the probability of having a complete/subtotal resection versus partial resection/biopsy for tumor site

1. Supratentorial midline
2. Cerebral hemispheres
3. Cerebellum

- **Supplementary figure 1**

**Legend:** Patient numbers for the various layers of analysis

- **Supplementary figure 2**

**Legend:** Extent of 1^st^ resection for 3440/4270 patients for interval from diagnosis to date of resection per time interval TI1-5: **A:** day 0-28; **B:** day 29-90; **C:** day 91-365; **D:** day >365

- **Supplementary figure 3**

**Legend:** Relative fraction of the type of initial biopsy for tumors of the supratentorial midline for intervention in the 1^st^ year following diagnosis per time interval T1-5

- **Supplementary figure 4**

**Legend:** Extent of resection and patient status at last follow-up for patients with
**→** incomplete 1^st^ resection (subtotal, partial resection, biopsy) and no further surgery or non-surgical therapy from groups TI2 to TI5 (n=666; patients without adequate follow-up excluded n=50) median follow-up 5.9 years (range 0.03-15.1 years).
Tumor site: cerebral hemispheres n=187, SML n=172, cerebellum n=197, caudal brainstem n=74, spinal cord n=36; and with
**→** complete 1^st^ resection and no further surgery or non-surgical therapy from groups TI2 to TI5 (n=899; patients without adequate follow-up excluded n=15) median follow-up 6.9 years (range 0.00-15.5 years).
Tumor site: cerebral hemispheres n=349, SML n=35, cerebellum n=493, caudal brainstem n=8, spinal cord n=14.

Abbreviations: CR: complete remission; SD: stable disease; PD: progressive disease; SML: supratentorial midline.

**Multivariable model building**

Binary logistic regression was used to analyze the simultaneous impact of the time interval (categorical: TI1-5) and further categorical explanatory variables on the extent of surgery. Extent of surgery was examined as complete resection, subtotal resection, partial resection, and biopsy. In all cases, odds were calculated as P/(1-P) with P being the ”Probability of the currently examined extent of resection”. Further explanatory variables were: age at diagnosis (categorical: <1yr, 1-4yrs, 5-9yrs, 10-15yrs, ≥16yrs), major tumor site (categorical: supratentorial midline (SML), cerebral hemispheres, cerebellum, caudal brainstem (BS), spinal cord), sex (binary: female, male), neurofibromatosis NF1 (binary: yes, no), and tuberous sclerosis complex (TSC) (binary: yes, no).

The model was built in three layers. In the first step, the variable “time interval” was included. In the second step, the further explanatory variables were put to the selection by means of forward selection (inclusion/exclusion criterion: p-value of Wald test ≤0.05/>0.1). The results of the main effects model (model after step two) are reported as odds ratio with 95% confidence interval and p-value of Wald test in Table 3. In a third step, the interaction of the further explanatory variables with “time interval” was offered for forward selection (inclusion criterion: p-value of Wald test ≤0.05). For explanatory variables with selected interaction with “time interval”, binary logistic main effects models were fitted in the subgroups defined by the (combination of) categories of these explanatory variables. Results are reported as odds ratio with 95% confidence interval and p-value of Wald test.

Additionally, multinomial logistic regression models were fitted to analyze the probability of having a complete or subtotal resection versus a partial resection or biopsy. Results are reported as odds ratio with 95% confidence interval and p-value of Wald test, with odds ratios calculated as P/Q with P being the ”Probability of total or subtotal resection” and Q being the ”Probability of partial resection or biopsy”.

| **Supplementary table 1 Extent of resection for interval between diagnosis and 1^st^ surgery (n=3440)** | | | | | |
| --- | --- | --- | --- | --- | --- |
| **Timing of 1^st^ tumor surgery and extent of resection** | **Group TI1**  **n=812 (%)** | **Group TI2**  **n=634 (%)** | **Group TI3**  **n=724 (%)** | **Group TI4**  **n=631 (%)** | **Group TI5**  **n=639 (%)** |
|  | | | | | |
| **within 28 days (n=2669)** | 658 (81.0%) | 510 (80.4%) | 544 (75.1%) | 453 (71.8%) | 504^a^ (78.9%) |
| - complete resection | 271 | 217 | 231 | 188 | 147 |
| - subtotal resection | 140 | 61 | 59 | 33 | 52 |
| - partial resection | 116 | 148 | 167 | 145 | 199 |
| - biopsy | 131 | 83 | 83 | 79 | 95 |
| - other^b^ | 0 | 1 | 4 | 8 | 11 |
|  | | | | | |
| **from 29 to 90 days (n=296)** | 77 (9.5%) | 45 (7.1%) | 64 (8.8%) | 62 (9.8%) | 48 (7.5%) |
| - complete resection | 27 | 14 | 21 | 25 | 13 |
| - subtotal resection | 18 | 2 | 5 | 4 | 1 |
| - partial resection | 14 | 15 | 17 | 22 | 17 |
| - biopsy | 18 | 13 | 21 | 11 | 16 |
| - other^b^ | 0 | 1 | 0 | 0 | 1 |
|  | | | | | |
| **from 91 to 365 days (n=235)** | 37 (4.6%) | 41 (6.5%) | 42 (5.8%) | 55 (8.7%) | 60 (9.4%) |
| - complete resection | 12 | 13 | 15 | 27 | 18 |
| - subtotal resection | 5 | 6 | 4 | 3 | 1 |
| - partial resection | 10 | 8 | 12 | 10 | 19 |
| - biopsy | 10 | 14 | 11 | 12 | 17 |
| - other^b^ | 0 | 0 | 0 | 3 | 5 |
|  | | | | | |
| **after >1 year (n=240)** | 40 (4.9%) | 38 (6.0%) | 74 (10.2%) | 61 (9.7%) | 27 (4.2%) |
| - complete resection | 9 | 9 | 25 | 20 | 8 |
| - subtotal resection | 0 | 4 | 4 | 0 | 0 |
| - partial resection | 14 | 6 | 17 | 18 | 7 |
| - biopsy | 17 | 19 | 27 | 22 | 11 |
| - other^b^ | 0 | 0 | 1 | 1 | 1 |
|  | | | | | |

a: including 1 patient operated for hydrocephalus with an inconclusive biopsy, but with radiologic tumor diagnosis on the early postoperative MRI; b: other interventions: surgery of metastases or extent of surgery not documented.

| **Supplementary table 2 Tumor localization for interval between diagnosis and 1^st^ surgery (n=3440)** | | | | | |
| --- | --- | --- | --- | --- | --- |
| **Timing of 1^st^ tumor surgery and tumor site** | **Group TI1**  **n=812 (%)** | **Group TI2**  **n=634 (%)** | **Group TI3**  **n=724 (%)** | **Group TI4**  **n=631 (%)** | **Group TI5**  **n=639 (%)** |
|  | | | | | |
| **within 28 days** | 658 (81.0%) | 510 (80.4%) | 544 (75.1%) | 453 (71.8%) | 504^a^ (78.9%) |
| - cerebral hemispheres | 120 | 119 | 134 | 98 | 107 |
| - supratentorial midline | 198 | 118 | 113 | 109 | 117 |
| - cerebellum | 257 | 214 | 230 | 202 | 216 |
| - caudal brainstem | 57 | 39 | 45 | 25 | 48 |
| - spinal cord | 26 | 20 | 22 | 19 | 15 |
| - disseminated | 0 | 0 | 0 | 0 | 1 |
|  | | | | | |
| **from 29 to 90 days** | 77 (9.5%) | 45 (7.1%) | 64 (8.8%) | 62 (9.8%) | 48 (7.5%) |
| - cerebral hemispheres | 30 | 17 | 31 | 39 | 27 |
| - supratentorial midline | 29 | 16 | 14 | 14 | 12 |
| - cerebellum | 7 | 6 | 11 | 4 | 3 |
| - caudal brainstem | 8 | 2 | 4 | 4 | 1 |
| - spinal cord | 3 | 4 | 0 | 1 | 5 |
| - disseminated | 0 | 0 | 0 | 0 | 0 |
|  | | | | | |
| **from 91 to 365 days** | 37 (4.6%) | 41 (6.5%) | 42 (5.8%) | 55 (8.7%) | 60 (9.4%) |
| - cerebral hemispheres | 20 | 19 | 24 | 31 | 39 |
| - supratentorial midline | 13 | 12 | 8 | 13 | 14 |
| - cerebellum | 3 | 6 | 4 | 6 | 4 |
| - caudal brainstem | 1 | 2 | 6 | 2 | 3 |
| - spinal cord | 0 | 1 | 4 | 1 | 0 |
| - disseminated | 0 | 0 | 0 | 2 | 0 |
|  | | | | | |
| **after >1 year** | 40 (4.9%) | 38 (6.0%) | 74 (10.2%) | 61 (9.7%) | 27 (4.2%) |
| - cerebral hemispheres | 7 | 8 | 25 | 24 | 14 |
| - supratentorial midline | 24 | 25 | 38 | 27 | 11 |
| - cerebellum | 7 | 2 | 9 | 8 | 1 |
| - caudal brainstem | 1 | 2 | 1 | 2 | 1 |
| - spinal cord | 1 | 1 | 1 | 0 | 0 |
| - disseminated | 0 | 0 | 0 | 0 | 0 |
|  | | | | | |

a: including 1 patient operated for hydrocephalus with an inconclusive biopsy, but with radiologic tumor diagnosis on the early postoperative MRI.

| **Supplementary table 3 Extent of 1^st^ resection for NF1-associated LGG for tumor site and time interval (n=628)** | | | | | |
| --- | --- | --- | --- | --- | --- |
| **Tumor site for NF1-associated LGG and**  **extent of 1^st^ resection** | **Group TI1**  **n= 115** | **Group TI2**  **n=112** | **Group TI3**  **n=143** | **Group TI4**  **n=133** | **Group TI5**  **n=125** |
|  | | | | | |
| **Cerebral hemispheres** | n=7 | n=1 | n=8 | n=9 | n=1 |
| surgery within 1^st^ year | 7 | 1 | 4 | 7 | 0 |
| - complete resection | 3 | 0 | 2 | 4 | 0 |
| - subtotal resection | 1 | 0 | 0 | 1 | 0 |
| - partial resection | 2 | 0 | 0 | 1 | 0 |
| - biopsy | 1 | 1 | 2 | 0 | 0 |
| - other^a^ | 0 | 0 | 0 | 1 | 0 |
| surgery delayed >1 year | 0 | 0 | 0 | 2 | 1 |
| follow-up <1 year | 0 | 0 | 0 | 0 | 0 |
| radiological diagnosis | 0 | 0 | 4 | 0 | 0 |
|  | | | | | |
| **Supratentorial midline** | n=100 | n=100 | n=116 | n=110 | n=102 |
| surgery within 1^st^ year | 19 | 5 | 8 | 7 | 11 |
| - complete resection | 0 | 0 | 0 | 2 | 0 |
| - subtotal resection | 3 | 0 | 1 | 0 | 0 |
| - partial resection | 8 | 2 | 3 | 1 | 0 |
| - biopsy | 8 | 3 | 4 | 4 | 10 |
| - other^a^ | 0 | 0 | 0 | 0 | 0 |
| surgery delayed >1 year | 10 | 12 | 14 | 5 | 3 |
| follow-up <1 year | 2 | 0 | 0 | 2 | 11 |
| radiological diagnosis | 69 | 83 | 94 | 96 | 77 |
|  | | | | | |
| **Cerebellum** | n=3 | n=8 | n=3 | n=3 | n=3 |
| surgery within 1^st^ year | 1 | 6 | 1 | 2 | 3 |
| - complete resection | 1 | 1 | 0 | 2 | 1 |
| - subtotal resection | 0 | 0 | 0 | 0 | 0 |
| - partial resection | 0 | 4 | 0 | 0 | 2 |
| - biopsy | 0 | 0 | 1 | 0 | 0 |
| - other^a^ | 0 | 1 | 0 | 0 | 0 |
| surgery delayed >1 year | 2 | 2 | 1 | 1 | 0 |
| follow-up <1 year | 0 | 0 | 0 | 0 | 0 |
| radiological diagnosis | 0 | 0 | 1 | 0 | 0 |
|  |  |  |  |  |  |
| **Caudal brainstem** | n=5 | n=2 | n=12 | n=9 | n=18 |
| surgery within 1^st^ year | 2 | 1 | 4 | 2 | 7 |
| - complete resection | 0 | 0 | 0 | 0 | 0 |
| - subtotal resection | 1 | 0 | 0 | 0 | 0 |
| - partial resection | 0 | 0 | 1 | 0 | 1 |
| - biopsy | 1 | 1 | 3 | 2 | 6 |
| - other^a^ | 0 | 0 | 0 | 0 | 0 |
| surgery delayed >1 year | 0 | 0 | 0 | 2 | 1 |
| follow-up <1 year | 0 | 0 | 1 | 0 | 2 |
| radiological diagnosis | 3 | 1 | 7 | 5 | 8 |
|  | | | | | |
| **Spinal cord** | n=0 | n=1 | n=4 | n=2 | n=1 |
| surgery within 1^st^ year | 0 | 1 | 2 | 2 | 1 |
| - complete resection | 0 | 1 | 1 | 1 | 0 |
| - subtotal resection | 0 | 0 | 0 | 0 | 0 |
| - partial resection | 0 | 0 | 1 | 1 | 0 |
| - biopsy | 0 | 0 | 0 | 0 | 1 |
| - other^a^ | 0 | 0 | 0 | 0 | 0 |
| surgery delayed >1 year | 0 | 0 | 0 | 0 | 0 |
| follow-up <1 year | 0 | 0 | 0 | 0 | 0 |
| radiological diagnosis | 0 | 0 | 2 | 0 | 0 |
|  | | | | | |

a: other interventions: surgery of metastases or extent of surgery not documented.

| **Supplementary table 4 Extent of 1^st^ resection for age group at diagnosis and tumor site per time interval (n=4270)** | | | | | |
| --- | --- | --- | --- | --- | --- |
| 1. **Age <1 year at diagnosis (n=211, 4.9%)** | | | | | |
| **Tumor site and**  **extent of 1^st^ resection** | **Group TI1**  **n=59** | **Group TI2**  **n=40** | **Group TI3**  **n=45** | **Group TI4**  **n=29** | **Group TI5**  **n=38** |
|  | | | | | |
| **Cerebral hemispheres** | n=11 | n=10 | n=13 | n=5 | n=10 |
| surgery within 1^st^ year | 11 | 5 | 8 | 5 | 8 |
| - complete resection | 4 | 0 | 5 | 1 | 3 |
| - subtotal resection | 4 | 1 | 0 | 1 | 0 |
| - partial resection | 2 | 2 | 2 | 2 | 5 |
| - biopsy | 1 | 2 | 1 | 1 | 0 |
| - other^a^ | 0 | 0 | 0 | 0 | 0 |
| surgery delayed >1 year | 0 | 3 | 2 | 0 | 0 |
| follow-up <1 year | 0 | 0 | 0 | 0 | 0 |
| radiological diagnosis | 0 | 2 | 3 | 0 | 2 |
|  | | | | | |
| **Supratentorial midline** | n=45 | n=22 | n=27 | n=21 | n=23 |
| surgery within 1^st^ year | 32 | 10 | 9 | 8 | 13 |
| - complete resection | 1 | 0 | 0 | 1 | 0 |
| - subtotal resection | 2 | 0 | 0 | 0 | 0 |
| - partial resection | 10 | 3 | 4 | 1 | 6 |
| - biopsy | 19 | 7 | 5 | 5 | 6 |
| - other^a^ | 0 | 0 | 0 | 1 | 1 |
| surgery delayed >1 year | 2 | 4 | 5 | 6 | 1 |
| follow-up <1 year | 2 | 1 | 0 | 2 | 1 |
| radiological diagnosis | 9 | 7 | 13 | 5 | 8 |
|  | | | | | |
| **Cerebellum** | n=2 | n=7 | n=2 | n=2 | n=4 |
| surgery within 1^st^ year | 2 | 7 | 2 | 2 | 4 |
| - complete resection | 0 | 0 | 1 | 2 | 1 |
| - subtotal resection | 0 | 2 | 1 | 0 | 0 |
| - partial resection | 1 | 3 | 0 | 0 | 2 |
| - biopsy | 1 | 2 | 0 | 0 | 1 |
| - other^a^ | 0 | 0 | 0 | 0 | 0 |
| surgery delayed >1 year | 0 | 0 | 0 | 0 | 0 |
| follow-up <1 year | 0 | 0 | 0 | 0 | 0 |
| radiological diagnosis | 0 | 0 | 0 | 0 | 0 |
|  | | | | | |
| **Caudal brainstem** | n=1 | n=1 | n=3 | n=0 | n=0 |
| surgery within 1^st^ year | 1 | 1 | 3 | 0 | 0 |
| - complete resection | 0 | 0 | 0 | 0 | 0 |
| - subtotal resection | 0 | 0 | 0 | 0 | 0 |
| - partial resection | 1 | 1 | 2 | 0 | 0 |
| - biopsy | 0 | 0 | 1 | 0 | 0 |
| - other^a^ | 0 | 0 | 0 | 0 | 0 |
| surgery delayed >1 year | 0 | 0 | 0 | 0 | 0 |
| follow-up <1 year | 0 | 0 | 0 | 0 | 0 |
| radiological diagnosis | 0 | 0 | 0 | 0 | 0 |
|  | | | | | |
| **Spinal cord** | n=0 | n=0 | n=0 | n=1 | n=1 |
| surgery within 1^st^ year | 0 | 0 | 0 | 1 | 1 |
| - complete resection | 0 | 0 | 0 | 0 | 1 |
| - subtotal resection | 0 | 0 | 0 | 0 | 0 |
| - partial resection | 0 | 0 | 0 | 0 | 0 |
| - biopsy | 0 | 0 | 0 | 1 | 0 |
| - other^a^ | 0 | 0 | 0 | 0 | 0 |
| surgery delayed >1 year | 0 | 0 | 0 | 0 | 0 |
| follow-up <1 year | 0 | 0 | 0 | 0 | 0 |
| radiological diagnosis | 0 | 0 | 0 | 0 | 0 |

| 1. **Age 1-4 years at diagnosis (n=1212, 28.4%)** | | | | | |
| --- | --- | --- | --- | --- | --- |
| **Tumor site and**  **extent of 1^st^ resection** | **Group TI1**  **n=288** | **Group TI2**  **n= 215** | **Group TI3**  **n=234** | **Group TI4**  **n=243** | **Group TI5**  **n=232** |
|  | | | | | |
| **Cerebral hemispheres** | n=28 | n=22 | n=22 | n=34 | n=30 |
| surgery within 1^st^ year | 27 | 16 | 17 | 26 | 25 |
| - complete resection | 8 | 5 | 6 | 10 | 7 |
| - subtotal resection | 8 | 3 | 4 | 0 | 1 |
| - partial resection | 6 | 6 | 7 | 11 | 11 |
| - biopsy | 5 | 2 | 0 | 4 | 5 |
| - other^a^ | 0 | 0 | 0 | 1 | 1 |
| surgery delayed >1 year | 1 | 2 | 1 | 7 | 3 |
| follow-up <1 year | 0 | 0 | 0 | 0 | 0 |
| radiological diagnosis | 0 | 4 | 4 | 1 | 2 |
|  | | | | | |
| **Supratentorial midline** | n=137 | n=97 | n=100 | n=121 | n=99 |
| surgery within 1^st^ year | 80 | 34 | 33 | 39 | 39 |
| - complete resection | 13 | 0 | 2 | 2 | 3 |
| - subtotal resection | 13 | 4 | 1 | 2 | 0 |
| - partial resection | 23 | 17 | 15 | 17 | 14 |
| - biopsy | 31 | 13 | 14 | 18 | 21 |
| - other^a^ | 0 | 0 | 1 | 0 | 1 |
| surgery delayed >1 year | 11 | 11 | 9 | 9 | 3 |
| follow-up <1 year | 0 | 0 | 0 | 2 | 8 |
| radiological diagnosis | 46 | 52 | 58 | 71 | 49 |
|  | | | | | |
| **Cerebellum** | n=88 | n=62 | n=82 | n=69 | n=68 |
| surgery within 1^st^ year | 85 | 59 | 76 | 65 | 67 |
| - complete resection | 55 | 30 | 48 | 43 | 33 |
| - subtotal resection | 16 | 12 | 10 | 6 | 7 |
| - partial resection | 10 | 16 | 14 | 15 | 22 |
| - biopsy | 4 | 1 | 4 | 1 | 2 |
| - other^a^ | 0 | 0 | 0 | 0 | 3 |
| surgery delayed >1 year | 2 | 0 | 3 | 3 | 0 |
| follow-up <1 year | 0 | 0 | 0 | 0 | 0 |
| radiological diagnosis | 1 | 3 | 3 | 1 | 1 |
|  | | | | | |
| **Caudal brainstem** | n=25 | n=22 | n=23 | n=12 | n=30 |
| surgery within 1^st^ year | 21 | 17 | 18 | 8 | 23 |
| - complete resection | 1 | 1 | 0 | 0 | 0 |
| - subtotal resection | 7 | 1 | 6 | 1 | 3 |
| - partial resection | 6 | 13 | 5 | 3 | 10 |
| - biopsy | 7 | 2 | 7 | 4 | 10 |
| - other^a^ | 0 | 0 | 0 | 0 | 0 |
| surgery delayed >1 year | 1 | 2 | 0 | 0 | 0 |
| follow-up <1 year | 0 | 0 | 1 | 0 | 3 |
| radiological diagnosis | 3 | 3 | 4 | 4 | 4 |
|  | | | | | |
| **Spinal cord** | n=10 | n=11 | n=7 | n=6 | n=4 |
| surgery within 1^st^ year | 9 | 11 | 7 | 5 | 4 |
| - complete resection | 4 | 1 | 0 | 1 | 0 |
| - subtotal resection | 0 | 3 | 0 | 1 | 1 |
| - partial resection | 4 | 5 | 7 | 3 | 3 |
| - biopsy | 1 | 2 | 0 | 0 | 0 |
| - other^a^ | 0 | 0 | 0 | 0 | 0 |
| surgery delayed >1 year | 1 | 0 | 0 | 0 | 0 |
| follow-up <1 year | 0 | 0 | 0 | 0 | 0 |
| radiological diagnosis | 0 | 0 | 0 | 1 | 0 |
|  | | | | | |
| **Primary dissemination**  type of surgery | n=0 | n=1  biopsy | n=0 | n=1  other^b^ | n=1  biopsy |
|  | | | | | |

| 1. **Age 5-9 years at diagnosis (n=1290, 30.2%)** | | | | | |
| --- | --- | --- | --- | --- | --- |
| **Tumor site and**  **extent of 1^st^ resection** | **Group TI1**  **n=292** | **Group TI2**  **n=248** | **Group TI3**  **n=298** | **Group TI4**  **n=214** | **Group TI5**  **n=238** |
|  | | | | | |
| **Cerebral hemispheres** | n=47 | n=38 | n=74 | n=45 | n=48 |
| surgery within 1^st^ year | 43 | 34 | 51 | 36 | 41 |
| - complete resection | 24 | 19 | 23 | 15 | 18 |
| - subtotal resection | 8 | 5 | 8 | 3 | 2 |
| - partial resection | 2 | 8 | 13 | 13 | 15 |
| - biopsy | 9 | 1 | 7 | 3 | 2 |
| - other^a^ | 0 | 1 | 0 | 2 | 4 |
| surgery delayed >1 year | 4 | 2 | 14 | 7 | 4 |
| follow-up <1 year | 0 | 0 | 0 | 0 | 1 |
| radiological diagnosis | 0 | 2 | 9 | 2 | 2 |
|  | | | | | |
| **Supratentorial midline** | n=101 | n=102 | n=103 | n=82 | n=86 |
| surgery within 1^st^ year | 67 | 51 | 36 | 39 | 38 |
| - complete resection | 6 | 7 | 4 | 3 | 2 |
| - subtotal resection | 14 | 2 | 2 | 1 | 2 |
| - partial resection | 17 | 11 | 11 | 16 | 12 |
| - biopsy | 30 | 31 | 19 | 19 | 21 |
| - other^a^ | 0 | 0 | 0 | 0 | 1 |
| surgery delayed >1 year | 6 | 5 | 15 | 4 | 4 |
| follow-up <1 year | 0 | 0 | 0 | 0 | 6 |
| radiological diagnosis | 28 | 46 | 52 | 39 | 38 |
|  | | | | | |
| **Cerebellum** | n=110 | n=88 | n=89 | n=62 | n=76 |
| surgery within 1^st^ year | 108 | 87 | 85 | 60 | 72 |
| - complete resection | 76 | 58 | 51 | 43 | 31 |
| - subtotal resection | 24 | 7 | 8 | 2 | 9 |
| - partial resection | 6 | 21 | 23 | 13 | 32 |
| - biopsy | 2 | 0 | 2 | 0 | 0 |
| - other^a^ | 0 | 1 | 1 | 2 | 0 |
| surgery delayed >1 year | 2 | 1 | 2 | 1 | 1 |
| follow-up <1 year | 0 | 0 | 0 | 0 | 1 |
| radiological diagnosis | 0 | 0 | 2 | 1 | 2 |
|  | | | | | |
| **Caudal brainstem** | n=27 | n=12 | n=19 | n=16 | n=19 |
| surgery within 1^st^ year | 26 | 11 | 15 | 13 | 15 |
| - complete resection | 0 | 1 | 0 | 0 | 0 |
| - subtotal resection | 10 | 1 | 2 | 1 | 0 |
| - partial resection | 10 | 3 | 8 | 6 | 7 |
| - biopsy | 6 | 6 | 5 | 6 | 8 |
| - other^a^ | 0 | 0 | 0 | 0 | 0 |
| surgery delayed >1 year | 0 | 0 | 1 | 0 | 1 |
| follow-up <1 year | 0 | 0 | 0 | 0 | 0 |
| radiological diagnosis | 1 | 1 | 3 | 3 | 3 |
|  | | | | | |
| **Spinal cord** | n=7 | n=8 | n=13 | n=9 | n=9 |
| surgery within 1^st^ year | 7 | 8 | 11 | 8 | 9 |
| - complete resection | 3 | 4 | 3 | 2 | 0 |
| - subtotal resection | 1 | 0 | 3 | 0 | 3 |
| - partial resection | 3 | 4 | 5 | 5 | 5 |
| - biopsy | 0 | 0 | 0 | 1 | 1 |
| - other^a^ | 0 | 0 | 0 | 0 | 0 |
| surgery delayed >1 year | 0 | 0 | 1 | 0 | 0 |
| follow-up <1 year | 0 | 0 | 0 | 0 | 0 |
| radiological diagnosis | 0 | 0 | 1 | 1 | 0 |
|  | | | | | |

| 1. **Age 10-15 years at diagnosis (n=1368, 32.1%)** | | | | | |
| --- | --- | --- | --- | --- | --- |
| **Tumor site and**  **extent of 1^st^ resection** | **Group TI1**  **n=272** | **Group TI2**  **n=250** | **Group TI3**  **n=296** | **Group TI4**  **n=287** | **Group TI5**  **n=263** |
|  | | | | | |
| **Cerebral hemispheres** | n=83 | n=93 | n=109 | n=100 | n=91 |
| surgery within 1^st^ year | 80 | 87 | 92 | 86 | 78 |
| - complete resection | 45 | 48 | 54 | 57 | 30 |
| - subtotal resection | 15 | 13 | 8 | 4 | 11 |
| - partial resection | 11 | 17 | 21 | 19 | 27 |
| - biopsy | 9 | 9 | 9 | 5 | 7 |
| - other^a^ | 0 | 0 | 0 | 1 | 3 |
| surgery delayed >1 year | 2 | 1 | 8 | 9 | 7 |
| follow-up <1 year | 0 | 0 | 0 | 0 | 1 |
| radiological diagnosis | 1 | 5 | 9 | 5 | 5 |
|  | | | | | |
| **Supratentorial midline** | n=87 | n=69 | n=81 | n=89 | n=80 |
| surgery within 1^st^ year | 59 | 49 | 48 | 40 | 44 |
| - complete resection | 10 | 6 | 3 | 0 | 1 |
| - subtotal resection | 11 | 4 | 4 | 3 | 0 |
| - partial resection | 16 | 16 | 19 | 13 | 15 |
| - biopsy | 22 | 23 | 21 | 23 | 28 |
| - other^a^ | 0 | 0 | 1 | 1 | 0 |
| surgery delayed >1 year | 5 | 5 | 8 | 8 | 3 |
| follow-up <1 year | 2 | 0 | 0 | 0 | 6 |
| radiological diagnosis | 21 | 15 | 25 | 41 | 27 |
|  | | | | | |
| **Cerebellum** | n=70 | n=67 | n=77 | n=76 | n=72 |
| surgery within 1^st^ year | 66 | 66 | 72 | 69 | 72 |
| - complete resection | 43 | 45 | 46 | 39 | 32 |
| - subtotal resection | 19 | 6 | 6 | 6 | 10 |
| - partial resection | 2 | 13 | 16 | 21 | 26 |
| - biopsy | 2 | 2 | 4 | 2 | 2 |
| - other^a^ | 0 | 0 | 0 | 1 | 2 |
| surgery delayed >1 year | 3 | 1 | 3 | 3 | 0 |
| follow-up <1 year | 0 | 0 | 0 | 0 | 0 |
| radiological diagnosis | 1 | 0 | 2 | 4 | 0 |
|  | | | | | |
| **Caudal brainstem** | n=19 | n=16 | n=19 | n=14 | n=14 |
| surgery within 1^st^ year | 17 | 14 | 17 | 9 | 13 |
| - complete resection | 2 | 5 | 1 | 0 | 0 |
| - subtotal resection | 5 | 1 | 4 | 2 | 1 |
| - partial resection | 5 | 5 | 7 | 3 | 7 |
| - biopsy | 5 | 3 | 5 | 4 | 5 |
| - other^a^ | 0 | 0 | 0 | 0 | 0 |
| surgery delayed >1 year | 0 | 0 | 0 | 2 | 0 |
| follow-up <1 year | 0 | 0 | 0 | 0 | 0 |
| radiological diagnosis | 2 | 2 | 2 | 3 | 1 |
|  | | | | | |
| **Spinal cord** | n=13 | n=5 | n=10 | n=7 | n=6 |
| surgery within 1^st^ year | 13 | 4 | 8 | 7 | 6 |
| - complete resection | 4 | 0 | 0 | 2 | 0 |
| - subtotal resection | 1 | 2 | 0 | 0 | 0 |
| - partial resection | 3 | 1 | 6 | 5 | 4 |
| - biopsy | 5 | 1 | 1 | 0 | 2 |
| - other^a^ | 0 | 0 | 1 | 0 | 0 |
| surgery delayed >1 year | 0 | 1 | 0 | 0 | 0 |
| follow-up <1 year | 0 | 0 | 0 | 0 | 0 |
| radiological diagnosis | 0 | 0 | 2 | 0 | 0 |
|  | | | | | |
| **Primary dissemination**  type of surgery | n=0 | n=0 | n=0 | n=1  other^b^ | n=0 |
|  | | | | | |

| 1. **Age ≥16 years at diagnosis (n=189, 4.4%)** | | | | | |
| --- | --- | --- | --- | --- | --- |
| **Tumor site and**  **extent of 1^st^ resection** | **Group TI1**  **n=18** | **Group TI2**  **n=27** | **Group TI3**  **n=52** | **Group TI4**  **n=47** | **Group TI5**  **n=45** |
|  | | | | | |
| **Cerebral hemispheres** | n=9 | n=14 | n=23 | n=16 | n=24 |
| surgery within 1^st^ year | 9 | 13 | 21 | 15 | 21 |
| - complete resection | 8 | 7 | 14 | 9 | 10 |
| - subtotal resection | 1 | 1 | 1 | 1 | 1 |
| - partial resection | 0 | 4 | 4 | 5 | 9 |
| - biopsy | 0 | 1 | 2 | 0 | 1 |
| - other^a^ | 0 | 0 | 0 | 0 | 0 |
| surgery delayed >1 year | 0 | 0 | 0 | 1 | 0 |
| follow-up <1 year | 0 | 0 | 0 | 0 | 0 |
| radiological diagnosis | 0 | 1 | 2 | 0 | 3 |
|  | | | | | |
| **Supratentorial midline** | n=2 | n=4 | n=13 | n=13 | n=11 |
| surgery within 1^st^ year | 2 | 2 | 9 | 10 | 9 |
| - complete resection | 0 | 0 | 1 | 1 | 1 |
| - subtotal resection | 0 | 1 | 0 | 1 | 1 |
| - partial resection | 2 | 1 | 4 | 3 | 2 |
| - biopsy | 0 | 0 | 4 | 5 | 5 |
| - other^a^ | 0 | 0 | 0 | 0 | 0 |
| surgery delayed >1 year | 0 | 0 | 1 | 0 | 0 |
| follow-up <1 year | 0 | 0 | 0 | 0 | 1 |
| radiological diagnosis | 0 | 2 | 3 | 3 | 1 |
|  |  |  |  |  |  |
| **Cerebellum** | n=6 | n=7 | n=13 | n=17 | n=8 |
| surgery within 1^st^ year | 6 | 7 | 10 | 16 | 8 |
| - complete resection | 3 | 6 | 5 | 8 | 5 |
| - subtotal resection | 3 | 0 | 0 | 5 | 2 |
| - partial resection | 0 | 0 | 2 | 3 | 0 |
| - biopsy | 0 | 1 | 3 | 0 | 0 |
| - other^a^ | 0 | 0 | 0 | 0 | 1 |
| surgery delayed >1 year | 0 | 0 | 1 | 1 | 0 |
| follow-up <1 year | 0 | 0 | 0 | 0 | 0 |
| radiological diagnosis | 0 | 0 | 2 | 0 | 0 |
|  | | | | | |
| **Caudal brainstem** | n=1 | n=0 | n=3 | n=1 | n=2 |
| surgery within 1^st^ year | 1 | 0 | 2 | 1 | 1 |
| - complete resection | 0 | 0 | 0 | 1 | 0 |
| - subtotal resection | 1 | 0 | 0 | 0 | 0 |
| - partial resection | 0 | 0 | 1 | 0 | 1 |
| - biopsy | 0 | 0 | 1 | 0 | 0 |
| - other^a^ | 0 | 0 | 0 | 0 | 0 |
| surgery delayed >1 year | 0 | 0 | 0 | 0 | 0 |
| follow-up <1 year | 0 | 0 | 0 | 0 | 1 |
| radiological diagnosis | 0 | 0 | 1 | 0 | 0 |
|  | | | | | |
| **Spinal cord** | n=0 | n=2 | n=0 | n=0 | n=0 |
| surgery within 1^st^ year | 0 | 2 | 0 | 0 | 0 |
| - complete resection | 0 | 1 | 0 | 0 | 0 |
| - subtotal resection | 0 | 0 | 0 | 0 | 0 |
| - partial resection | 0 | 1 | 0 | 0 | 0 |
| - biopsy | 0 | 0 | 0 | 0 | 0 |
| - other^a^ | 0 | 0 | 0 | 0 | 0 |
| surgery delayed >1 year | 0 | 0 | 0 | 0 | 0 |
| follow-up <1 year | 0 | 0 | 0 | 0 | 0 |
| radiological diagnosis | 0 | 0 | 0 | 0 | 0 |
|  | | | | | |

a: other interventions: surgery of metastases or extent of surgery not documented.

| **Supplementary table 5 Extent of 1^st^ resection for main histologic subgroups per time interval (n=4270)** | | | | | |
| --- | --- | --- | --- | --- | --- |
| **Histologic subgroup for extent of 1^st^ resection** | **Group TI1**  **n=929** | **Group TI2**  **n=780** | **Group TI3**  **n=925** | **Group TI4**  **n=820** | **Group TI5**  **n=816** |
|  | | | | | |
| **Pilocytic astrocytoma^a^** | n=629 | n=426 | n=457 | n=385 | n=401 |
| surgery within 1^st^ year | 600 | 403 | 423 | 359 | 393 |
| - complete resection | 234 | 167 | 188 | 154 | 111 |
| - subtotal resection | 135 | 49 | 49 | 27 | 40 |
| - partial resection | 112 | 121 | 129 | 122 | 166 |
| - biopsy | 119 | 66 | 53 | 50 | 67 |
| - other^b^ | 0 | 0 | 4 | 6 | 9 |
| surgery delayed >1 year | 29 | 23 | 34 | 26 | 8 |
|  | | | | | |
| **Diffuse glioma WHOII^c^** | n=56 | n=55 | n=58 | n=31 | n=27 |
| surgery within 1^st^ year | 54 | 52 | 52 | 27 | 26 |
| - complete resection | 14 | 14 | 11 | 6 | 6 |
| - subtotal resection | 9 | 4 | 5 | 2 | 1 |
| - partial resection | 9 | 15 | 12 | 7 | 8 |
| - biopsy | 22 | 19 | 24 | 12 | 11 |
| - other^b^ | 0 | 0 | 0 | 0 | 0 |
| surgery delayed >1 year | 2 | 3 | 6 | 4 | 1 |
|  | | | | | |
| **Glioneuronal LGG^d^** | n=83 | n=104 | n=128 | n=136 | n=130 |
| surgery within 1^st^ year | 79 | 98 | 114 | 118 | 120 |
| - complete resection | 42 | 45 | 47 | 61 | 41 |
| - subtotal resection | 13 | 13 | 10 | 7 | 8 |
| - partial resection | 16 | 26 | 40 | 34 | 48 |
| - biopsy | 8 | 13 | 17 | 15 | 16 |
| - other^b^ | 0 | 1 | 0 | 1 | 7 |
| surgery delayed >1 year | 4 | 6 | 14 | 18 | 10 |
|  | | | | | |
| **Other LGG^e^** | n=36 | n=42 | n=63 | n=65 | n=66 |
| surgery within 1^st^ year | 33 | 39 | 48 | 55 | 61 |
| - complete resection | 18 | 18 | 21 | 18 | 18 |
| - subtotal resection | 5 | 3 | 4 | 4 | 5 |
| - partial resection | 3 | 8 | 13 | 13 | 13 |
| - biopsy | 7 | 9 | 10 | 16 | 24 |
| - other^b^ | 0 | 1 | 0 | 4 | 1 |
| surgery delayed >1 year | 3 | 3 | 15 | 10 | 5 |
|  | | | | | |
| **All other patients** | n=125 | n=153 | n=219 | n=203 | n=192 |
| surgery within 1^st^ year^f^ | 6 | 4 | 13 | 11 | 12 |
| - complete resection | 2 | 0 | 0 | 1 | 2 |
| - subtotal resection | 1 | 0 | 0 | 0 | 0 |
| - partial resection | 0 | 1 | 2 | 1 | 0 |
| - biopsy | 3 | 3 | 11 | 9 | 10 |
| - other^b^ | 0 | 0 | 0 | 0 | 0 |
| surgery delayed >1 year^f^ | 2 | 3 | 5 | 3 | 3 |
| observation <1 year | 4 | 1 | 1 | 4 | 29 |
| radiological diagnosis | 113 | 145 | 200 | 185 | 148 |
|  | | | | | |

**a:** including pilomyxoid astrocytoma in TI 2 (n=6), 3 (n=13), 4 (n=8), and 5 (n=7);
**b**: other interventions: surgery of metastases or extent of surgery not documented;
**c:** comprising astrocytoma WHO grade II (n=198), oligodendroglioma WHO grade II (n=16), oligo-astrocytoma WHO grade II (n=13);  **d:** comprising desmoplastic infantile ganglioglioma/astrocytoma (n=32), ganglioglioma/PLNTY (n=381), dysembryoplastic neuro-epithelial tumor (n=148), rosette forming glioneuronal tumor (n=17), papillary glioneuronal tumor (n=3);
**e:** comprising subependymal giant cell astrocytoma WHO grade I (n=47), angiocentric glioma WHO grade I (n=17), LGG not otherwise specified (n=78), pleomorphic xanthoastrocytoma WHO grade II (n=49);
**f:** no tumor tissue in pathologic specimen n=56, not documented n=3, anaplastic astrocytoma WHO grade 3 in biopsy following 2, 6 and 12 years after clinical diagnosis of LGG n=3.

| **Supplementary table 6 Univariate analysis of the distribution of epidemiologic data** | | | | | | | | | | |
| --- | --- | --- | --- | --- | --- | --- | --- | --- | --- | --- |
| 1. **Median patient age at diagnosis for time interval (n=4231)** (p<0.001, Kruskal-Wallis test) | | | | | | | | | | |
| Time interval | | Median age (years) | | Range (minimum-maximum, years) | | | | | Skewness | |
| 1 | | 6.995 | | 0.0438 | | | 16.950 | | 0.248 | |
| 2 | | 7.608 | | 0.1095 | | | 17.613 | | 0.188 | |
| 3 | | 7.976 | | 0.0109 | | | 17.892 | | 0.137 | |
| 4 | | 8.038 | | 0.2464 | | | 17.944 | | 0.129 | |
| 5 | | 7.584 | | 0.0136 | | | 17.823 | | 0.175 | |
| all | | 7.581 | | 0.0109 | | | 17.944 | | 0.186 | |
|  | | | | | | | | | | |
| 1. **Distribution of age groups for time interval (n=4231)** (p<0.001, Chi^2^-test) | | | | | | | | | | |
|  | **TI 1** | | **TI 2** | | **TI 3** | **TI 4** | | **TI 5** | | **All** |
| <1 year  (% of TI) | 57 | | 39 | | 45 | 27 | | 37 | | 205 |
|  | 6,2% | | 5,0% | | 4,9% | 3,3% | | 4,7% | | 4,8% |
| 1-4 years  (% of TI) | 288 | | 215 | | 233 | 241 | | 221 | | 1198 |
|  | 31,1% | | 27,6% | | 25,2% | 29,5% | | 28,1% | | 28,3% |
| 5-9 years  (% of TI) | 292 | | 248 | | 298 | 214 | | 230 | | 1282 |
|  | 31,6% | | 31,8% | | 32,3% | 26,2% | | 29,2% | | 30,3% |
| 10-15 years  (% of TI) | 270 | | 250 | | 296 | 287 | | 256 | | 1359 |
|  | 29,2% | | 32,1% | | 32,0% | 35,2% | | 32,5% | | 32,1% |
| ≥16 years  (% of TI) | 18 | | 27 | | 52 | 47 | | 43 | | 187 |
|  | 1,9% | | 3,5% | | 5,6% | 5,8% | | 5,5% | | 4,4% |
| All  (% of TI) | 925 | | 779 | | 924 | 816 | | 787 | | 4231 |
|  | 100,0% | | 100,0% | | 100,0% | 100,0% | | 100,0% | | 100,0% |
|  | | | | | | | | | | |
| 1. **Distribution of male/female patients for time interval (n=4231)** (p=0.812 Chi^2^-test) | | | | | | | | | | |
|  | **TI 1** | | **TI 2** | | **TI 3** | **TI 4** | | **TI 5** | | **All** |
| Male  (% of TI) | 486 | | 421 | | 485 | 433 | | 401 | | 2226 |
|  | 52.5% | | 54.0% | | 52.5% | 53.1% | | 51.0% | | 52.6% |
| Female  (% of TI) | 439 | | 358 | | 439 | 383 | | 386 | | 2005 |
|  | 47.5% | | 46.0% | | 47.5% | 46.9% | | 49.0% | | 47.4% |
| All  (% of TI) | 925 | | 779 | | 924 | 816 | | 787 | | 4231 |
|  | 100.0% | | 100.0% | | 100.0% | 100.0% | | 100.0% | | 100.0% |
|  | | | | | | | | | | |
| 1. **NF1 status for time interval (n=4231)** (p=0.192 Chi^2^-test) | | | | | | | | | | |
|  | **TI 1** | | **TI 2** | | **TI 3** | **TI 4** | | **TI 5** | | **All** |
| No NF1  (% of TI) | 812 | | 667 | | 782 | 685 | | 675 | | 3621 |
|  | 87.8% | | 85.6% | | 84.6% | 83.9% | | 85.8% | | 85.6% |
| NF1  (% of TI) | 113 | | 112 | | 142 | 131 | | 112 | | 610 |
|  | 12.2% | | 14.4% | | 15.4% | 16.1% | | 14.2% | | 14.4% |
| All  (% of TI) | 925 | | 779 | | 924 | 816 | | 787 | | 4231 |
|  | 100.0% | | 100.0% | | 100.0% | 100.0% | | 100.0% | | 100.0% |
|  | | | | | | | | | | |
| 1. **TSC status for time interval (n=4231)** (p=0.016, Chi^2^-test) | | | | | | | | | | |
|  | **TI 1** | | **TI 2** | | **TI 3** | **TI 4** | | **TI 5** | | **All** |
| No TSC  (% of TI) | 913 | | 763 | | 906 | 810 | | 783 | | 4175 |
|  | 98.7% | | 97.9% | | 98.1% | 99.3% | | 99.5% | | 98.7% |
| TSC  (% of TI) | 12 | | 16 | | 18 | 6 | | 4 | | 56 |
|  | 1.3% | | 2.1% | | 1.9% | 0.7% | | 0.5% | | 1.3% |
| All  (% of TI) | 925 | | 779 | | 924 | 816 | | 787 | | 4231 |
|  | 100.0% | | 100.0% | | 100.0% | 100.0% | | 100.0% | | 100.0% |
|  | | | | | | | | | | |
| 1. **Tumor site for time interval (n=4227, n=4 multifocal primary excluded)** (p=0.090, Chi^2^-test) | | | | | | | | | | |
|  | **TI 1** | | **TI 2** | | **TI 3** | **TI 4** | | **TI 5** | | **All** |
| Cerebral H.  (% of TI) | 178 | | 177 | | 241 | 200 | | 201 | | 997 |
|  | 19.2% | | 22.8% | | 26.1% | 24.6% | | 25.6% | | 23.6% |
| SML  (% of TI) | 368 | | 293 | | 324 | 322 | | 277 | | 1584 |
|  | 39.8% | | 37.7% | | 35.1% | 39.6% | | 35.2% | | 37.5% |
| Cerebellum  (% of TI) | 276 | | 231 | | 263 | 226 | | 227 | | 1223 |
|  | 29.8% | | 29.7% | | 28.5% | 27.8% | | 28.9% | | 28.9% |
| Caudal BS  (% of TI) | 73 | | 51 | | 66 | 43 | | 61 | | 294 |
|  | 7.9% | | 6.6% | | 7.1% | 5.3% | | 7.8% | | 7.0% |
| Spinal cord  (% of TI) | 30 | | 26 | | 30 | 23 | | 20 | | 129 |
|  | 3.2% | | 3.3% | | 3.2% | 2.8% | | 2.5% | | 3.1% |
| All  (% of TI) | 925 | | 778 | | 924 | 814 | | 786 | | 4227 |
|  | 100.0% | | 100.0% | | 100.0% | 100.0% | | 100.0% | | 100.0% |
|  | | | | | | | | | | |
| 1. **Tumor dissemination for time interval (n=4231)** (p=0.706, Chi^2^-test) | | | | | | | | | | |
|  | **TI 1** | | **TI 2** | | **TI 3** | **TI 4** | | **TI 5** | | **All** |
| No*  (% of TI) | 882 | | 748 | | 878 | 785 | | 749 | | 4042 |
|  | 95.4% | | 96.0% | | 95.0% | 96.2% | | 95.2% | | 95.5% |
| Yes  (% of TI) | 43 | | 31 | | 46 | 31 | | 38 | | 189 |
|  | 4.6% | | 4.0% | | 5.0% | 3.8% | | 4.8% | | 4.5% |
| All  (% of TI) | 925 | | 779 | | 924 | 816 | | 787 | | 4231 |
|  | 100.0% | | 100.0% | | 100.0% | 100.0% | | 100.0% | | 100.0% |
|  | | | | | | | | | | |

Abbreviations: Cerebral H.: cerebral hemispheres; SML: supratentorial midline; BS: brainstem; NF1: neurofibromatosis type 1; TSC: tuberous sclerosis complex; * including 6 patients for whom tumor dissemination was questionable/not known.

| **Supplementary table 7 Results of binary logistic regression analysis examining the extent of neurosurgical resection for tumor site** | | | | | |
| --- | --- | --- | --- | --- | --- |
| 1. **Supratentorial midline (n=1584)** | | | | | |
| **Parameter** | | **Odds-Ratio*** | **(95%-CI)** | | **P-value**** |
| 1. **complete resection** | | | | | |
| Time Interval | |  |  |  | 0.003 |
|  | 2 vs 1 | 0.573 | 0.291 | 1.128 | 0.107 |
|  | 3 vs 1 | 0.400 | 0.191 | 0.837 | 0.015 |
|  | 4 vs 1 | 0.269 | 0.116 | 0.625 | 0.002 |
|  | 5 vs 1 | 0.311 | 0.134 | 0.724 | 0.007 |
| Age at diagnosis | | - - - | - - - | - - - | 0.211 |
| NF 1: no vs yes | | 15.660 | 3.814 | 64.304 | <0.001 |
| 1. **subtotal resection** | | | | | |
| Time Interval | |  |  |  | <0.001 |
|  | 2 vs 1 | 0.344 | 0.172 | 0.686 | 0.002 |
|  | 3 vs 1 | 0.198 | 0.087 | 0.450 | <0.001 |
|  | 4 vs 1 | 0.193 | 0.085 | 0.440 | <0.001 |
|  | 5 vs 1 | 0.094 | 0.029 | 0.308 | <0.001 |
| Age at diagnosis | | - - - | - - - | - - - | 0.113 |
| NF 1: no vs yes | | 7.434 | 2.680 | 20.621 | <0.001 |
| 1. **partial resection** | | | | | |
| Time Interval | |  |  |  | 0.945 |
|  | 2 vs 1 | 0.975 | 0.638 | 1.489 | 0.906 |
|  | 3 vs 1 | 0.992 | 0.655 | 1.501 | 0.969 |
|  | 4 vs 1 | 0.866 | 0.570 | 1.317 | 0.502 |
|  | 5 vs 1 | 1.042 | 0.681 | 1.593 | 0.851 |
| Age at diagnosis | |  |  |  | 0.046 |
|  | 1-4 years vs < 1 year | 1.832 | 1.096 | 3.061 | 0.021 |
|  | 5-9 years vs < 1 year | 1.145 | 0.681 | 1.925 | 0.609 |
|  | 10-15 years vs < 1 year | 1.357 | 0.813 | 2.263 | 0.243 |
|  | ≥ 16 years vs < 1 year | 1.961 | 0.866 | 4.442 | 0.106 |
| NF 1: no vs yes | | 12.478 | 7.110 | 21.900 | <0.001 |
| 1. **biopsy** | | | | | |
| Time Interval | |  |  |  | 0.057 |
|  | 2 vs 1 | 0.985 | 0.683 | 1.421 | 0.936 |
|  | 3 vs 1 | 0.700 | 0.482 | 1.016 | 0.061 |
|  | 4 vs 1 | 0.786 | 0.545 | 1.133 | 0.197 |
|  | 5 vs 1 | 1.207 | 0.839 | 1.738 | 0.311 |
| Age at diagnosis | | - - - | - - - | - - - | 0.780 |
| NF 1: no vs yes | | 8.463 | 5.693 | 12.580 | <0.001 |
| 1. **Cerebral hemispheres (n=997)** | | | | | |
| **Parameter** | | **Odds-Ratio*** | **(95%-CI)** | | **P-value**** |
| 1. **complete resection** | | | | | |
| Time Interval | |  |  |  | 0.010 |
|  | 2 vs 1 | 0.746 | 0.487 | 1.143 | 0.178 |
|  | 3 vs 1 | 0.675 | 0.453 | 1.006 | 0.054 |
|  | 4 vs 1 | 0.805 | 0.532 | 1.218 | 0.304 |
|  | 5 vs 1 | 0.470 | 0.307 | 0.719 | <0.001 |
| Age at diagnosis | |  |  |  | <0.001 |
|  | 1-4 years vs < 1 year | 0.988 | 0.468 | 2.083 | 0.974 |
|  | 5-9 years vs < 1 year | 1.820 | 0.915 | 3.620 | 0.088 |
|  | 10-15 years vs < 1 year | 2.716 | 1.398 | 5.278 | 0.003 |
|  | ≥ 16 years vs < 1 year | 3.796 | 1.755 | 8.211 | <0.001 |
| NF 1: no vs yes | | - - - | - - - | - - - | 0.154 |
| 1. **subtotal resection** | | | | | |
| Time Interval | |  |  |  | <0.001 |
|  | 2 vs 1 | 0.589 | 0.333 | 1.042 | 0.069 |
|  | 3 vs 1 | 0.377 | 0.211 | 0.671 | <0.001 |
|  | 4 vs 1 | 0.186 | 0.087 | 0.398 | <0.001 |
|  | 5 vs 1 | 0.318 | 0.168 | 0.604 | <0.001 |
| Age at diagnosis | | - - - | - - - | - - - | 0.798 |
| NF 1: no vs yes | | - - - | - - - | - - - | 0.641 |
| 1. **partial resection** | | | | | |
| Time Interval | |  |  |  | <0.001 |
|  | 2 vs 1 | 1.976 | 1.104 | 3.536 | 0.022 |
|  | 3 vs 1 | 1.811 | 1.039 | 3.158 | 0.036 |
|  | 4 vs 1 | 2.492 | 1.428 | 4.348 | 0.001 |
|  | 5 vs 1 | 3.738 | 2.175 | 6.425 | <0.001 |
| Age at diagnosis | | - - - | - - - | - - - | 0.125 |
| NF 1: no vs yes | | - - - | - - - | - - - | 0.334 |
| 1. **biopsy** | | | | | |
| Time Interval | |  |  |  | 0.147 |
|  | 2 vs 1 | 0.594 | 0.300 | 1.175 | 0.134 |
|  | 3 vs 1 | 0.549 | 0.291 | 1.037 | 0.065 |
|  | 4 vs 1 | 0.446 | 0.220 | 0.905 | 0.025 |
|  | 5 vs 1 | 0.517 | 0.262 | 1.021 | 0.057 |
| Age at diagnosis | | - - - | - - - | - - - | 0.507 |
| NF 1: no vs yes | | - - - | - - - | - - - | 0.518 |
| 1. **Cerebellum (n=1223)** | | | | | |
| **Parameter** | | **Odds-Ratio*** | **(95%-CI)** | | **P-value**** |
| 1. **complete resection** | | | | | |
| Time Interval | |  |  |  | <0.001 |
|  | 2 vs 1 | 0.907 | 0.629 | 1.308 | 0.601 |
|  | 3 vs 1 | 0.763 | 0.538 | 1.083 | 0.130 |
|  | 4 vs 1 | 0.857 | 0.594 | 1.237 | 0.410 |
|  | 5 vs 1 | 0.465 | 0.324 | 0.668 | <0.001 |
| Age at diagnosis | |  |  |  | 0.047 |
|  | 1-4 years vs < 1 year | 4.263 | 1.350 | 13.460 | 0.013 |
|  | 5-9 years vs < 1 year | 5.211 | 1.654 | 16.414 | 0.005 |
|  | 10-15 years vs < 1 year | 4.494 | 1.423 | 14.197 | 0.010 |
|  | ≥ 16 years vs < 1 year | 3.674 | 1.042 | 12.961 | 0.043 |
| NF 1: no vs yes | | 4.615 | 1.648 | 12.927 | 0.004 |
| 1. **sutotal resection** | | | | | |
| Time Interval | |  |  |  | <0.001 |
|  | 2 vs 1 | 0.457 | 0.280 | 0.746 | 0.002 |
|  | 3 vs 1 | 0.363 | 0.220 | 0.598 | <0.001 |
|  | 4 vs 1 | 0.317 | 0.183 | 0.548 | <0.001 |
|  | 5 vs 1 | 0.486 | 0.299 | 0.790 | 0.004 |
| Age at diagnosis | | - - - | - - - | - - - | 0.248 |
| NF 1: no vs yes | | - - - | - - - | - - - | 0.998 |
| 1. **partial resection** | | | | | |
| Time Interval | |  |  |  | <0.001 |
|  | 2 vs 1 | 4.027 | 2.306 | 7.036 | <0.001 |
|  | 3 vs 1 | 3.577 | 2.058 | 6.216 | <0.001 |
|  | 4 vs 1 | 4.042 | 2.310 | 7.074 | <0.001 |
|  | 5 vs 1 | 7.649 | 4.462 | 13.113 | <0.001 |
| Age at diagnosis | | - - - | - - - | - - - | 0.152 |
| NF 1: no vs yes | | - - - | - - - | - - - | 0.227 |
| 1. **biopsy** | | | | | |
| Time Interval | |  |  |  | 0.119 |
|  | 2 vs 1 | 0.621 | 0.208 | 1.850 | 0.392 |
|  | 3 vs 1 | 1.396 | 0.576 | 3.381 | 0.460 |
|  | 4 vs 1 | 0.313 | 0.082 | 1.196 | 0.089 |
|  | 5 vs 1 | 0.551 | 0.178 | 1.708 | 0.302 |
| Age at diagnosis | |  |  |  | <0.001 |
|  | 1-4 years vs < 1 year | 0.091 | 0.025 | 0.338 | <0.001 |
|  | 5-9 years vs < 1 year | 0.025 | 0.005 | 0.117 | <0.001 |
|  | 10-15 years vs < 1 year | 0.097 | 0.026 | 0.358 | <0.001 |
|  | ≥ 16 years vs < 1 year | 0.256 | 0.053 | 1.229 | 0.089 |
| NF 1: no vs yes | | - - - | - - - | - - - | 0.378 |

* Odds-Ratio of selected variables in the final model. Odds calculated as P/(1-P) with P= “Probability of the currently examined extent of resection”. ** P-value of Wald test in final model / step of removal for selected / non-selected variables

| **Supplementary table 8 Results of multi-nominal logistic regression analysis examining the probability of having a complete/subtotal resection versus partial resection/biopsy for tumor site** | | | | | |
| --- | --- | --- | --- | --- | --- |
| 1. **Supratentorial midline** | | | | | |
| **Parameter** | | **Odds-Ratio** | **(95%-CI)** | | **P-value**** |
| Time Interval | |  |  |  | <0.001 |
|  | 2 vs 1 | 0.445 | 0.263 | 0.750 | 0.002 |
|  | 3 vs 1 | 0.323 | 0.179 | 0.583 | <0.001 |
|  | 4 vs 1 | 0.253 | 0.135 | 0.474 | <0.001 |
|  | 5 vs 1 | 0.170 | 0.084 | 0.346 | <0.001 |
| Age at diagnosis | |  |  |  | <0.001 |
|  | 1-4 years vs < 1 year | 4.027 | 1.376 | 11.791 | 0.011 |
|  | 5-9 years vs < 1 year | 4.537 | 1.552 | 13.265 | 0.006 |
|  | 10-15 years vs < 1 year | 4.462 | 1.524 | 13.064 | 0.006 |
|  | ≥ 16 years vs < 1 year | 7.232 | 1.820 | 28.734 | 0.005 |
| NF 1: no vs yes | | - - - | - - - | - - - | 0.217 |
| 1. **Cerebral hemispheres** | | | | | |
| **Parameter** | | **Odds-Ratio** | **(95%-CI)** | | **P-value**** |
| Time Interval | |  |  |  | <0.001 |
|  | 2 vs 1 | 0.661 | 0.407 | 1.072 | 0.093 |
|  | 3 vs 1 | 0.616 | 0.388 | 0.976 | 0.039 |
|  | 4 vs 1 | 0.545 | 0.340 | 0.873 | 0.012 |
|  | 5 vs 1 | 0.341 | 0.214 | 0.543 | <0.001 |
| Age at diagnosis | |  |  |  | <0.001 |
|  | 1-4 years vs < 1 years | 0.903 | 0.423 | 1.927 | 0.791 |
|  | 5-9 years vs < 1 year | 1.694 | 0.827 | 3.470 | 0.150 |
|  | 10-15 years vs < 1 year | 2.155 | 1.083 | 4.287 | 0.029 |
|  | ≥ 16 years vs < 1 year | 2.265 | 1.006 | 5.101 | 0.048 |
| NF 1: no vs yes | | - - - | - - - | - - - | 0.468 |
| 1. **Cerebellum** | | | | | |
| **Parameter** | | **Odds-Ratio** | **(95%-CI)** | | **P-value**** |
| Time Interval | |  |  |  | <0.001 |
|  | 2 vs 1 | 0.340 | 0.208 | 0.557 | <0.001 |
|  | 3 vs 1 | 0.301 | 0.186 | 0.488 | <0.001 |
|  | 4 vs 1 | 0.326 | 0.198 | 0.537 | <0.001 |
|  | 5 vs 1 | 0.174 | 0.108 | 0.281 | <0.001 |
| Age at diagnosis*** | | - - - | - - - | - - - | 0.430 |
| NF 1: no vs yes | | 4.309 | 1.325 | 14.015 | 0.015 |

* Odds-Ratio of selected variables in the final model. Odds calculated as P/Q with P= “Probability of total or subtotal resection” and Q = “Probability of partial resection or biopsy”.

** P-value of Wald test in final model / step of removal for selected / non-selected variables

*** Because of too small numbers, categories “< 1 year” and “1-4 years” were combined for this analysis to ensure model convergence.

**Supplementary figure 1**

All patients n=4317

German LGG studies

Epidemiologic data and surgery per time interval

None

N=4270

- complete cohort n=4270
- at least 1 tumor-related surgery n=3440

Study population n=4270

date of diagnosis 01.10.1996-31.12.2018

Exclusion n=47

date of diagnosis before 01.10.1996 (Start of HIT-LGG 1996)

n=4317

Inclusion

Exclusion

N=39

- No surgery, observation <1 year after diagnosis

N=1070

- Surgery beyond the 1^st^ year of diagnosis n=240
- No surgery, observation ≥1 year after diagnosis n=791
- No surgery, observation <1 year after diagnosis n=39

N=830

- No surgery, observation ≥1 year after diagnosis n=791
- No surgery, observation <1 year after diagnosis n=39

N=4231

- 1^st^ surgery within the 1^st^ year of diagnosis n=3200
- Observation of at least 1 year after diagnosis, no surgery n=791
- Observation within 1^st^ year, 1^st^ surgery beyond the 1^st^ year of diagnosis n=240

N=3200

- 1^st^ surgery within the 1^st^ year of diagnosis

N=3440

- 1^st^ surgery within the 1^st^ year of diagnosis n=3200
- 1^st^ surgery beyond the 1^st^ year of diagnosis n=240

Descriptive analysis of extent of resection for 1^st^ surgery per time interval

Descriptive analysis of extent of resection for 1^st^ surgery within the 1^st^ year per time interval

Statistical analysis of extent of resection for 1^st^ surgery within the 1^st^ year per time interval

**Supplementary figure 2**

**Supplementary figure 3**

**Supplementary figure 4**


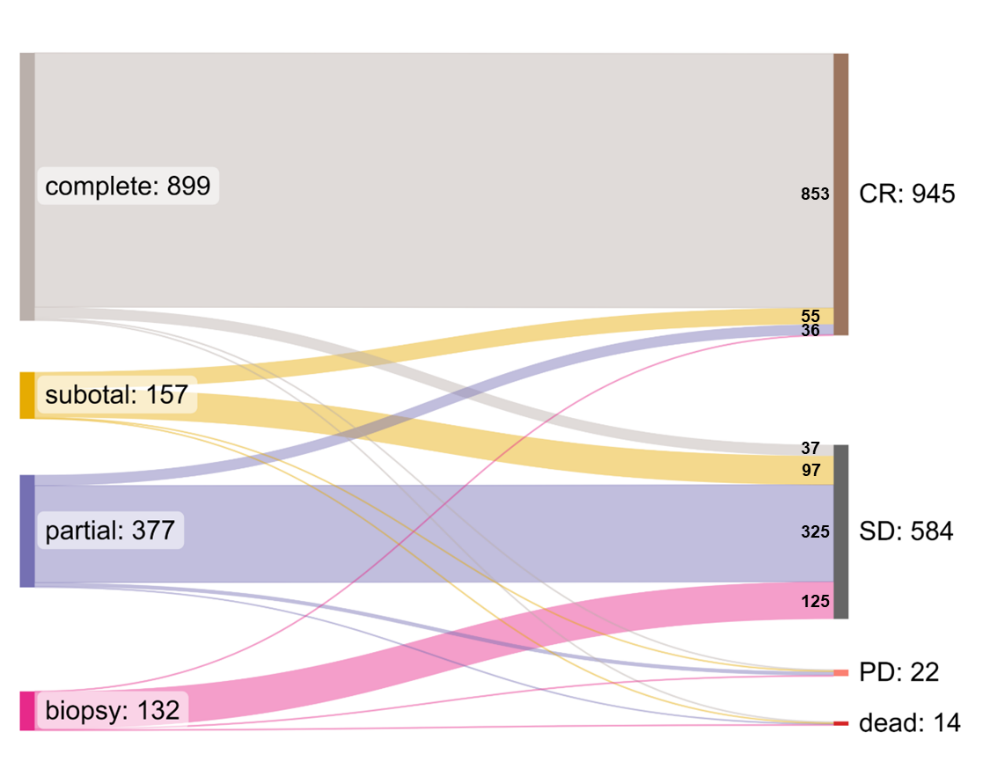

Supplement: Supplementary file 1 — Data S1. [file CAM4-13-e7417-s001.docx]
